# Prokaryotic Argonaute from *Archaeoglobus fulgidus* interacts with DNA as a homodimer

**DOI:** 10.1038/s41598-021-83889-4

**Published:** 2021-02-25

**Authors:** Edvardas Golovinas, Danielis Rutkauskas, Elena Manakova, Marija Jankunec, Arunas Silanskas, Giedrius Sasnauskas, Mindaugas Zaremba

**Affiliations:** 1 Institute of Biotechnology, Life Sciences Center, Vilnius University, Sauletekio av. 7, 10257 Vilnius, Lithuania; 2 Institute of Physics, Center for Physical Sciences and Technology, Savanoriu 231, 02300 Vilnius, Lithuania; 3 Institute of Biochemistry, Life Sciences Center, Vilnius University, Sauletekio av. 7, 10257 Vilnius, Lithuania

**Keywords:** Biochemistry, Biophysics, Molecular biology, Structural biology

## Abstract

Argonaute (Ago) proteins are found in all three domains of life. The best-characterized group is eukaryotic Argonautes (eAgos), which are the core of RNA interference. The best understood prokaryotic Ago (pAgo) proteins are full-length pAgos. They are composed of four major structural/functional domains (N, PAZ, MID, and PIWI) and thereby closely resemble eAgos. It was demonstrated that full-length pAgos function as prokaryotic antiviral systems, with the PIWI domain performing cleavage of invading nucleic acids. However, the majority of identified pAgos are shorter and catalytically inactive (encode just MID and inactive PIWI domains), thus their action mechanism and function remain unknown. In this work we focus on AfAgo, a short pAgo protein encoded by an archaeon *Archaeoglobus fulgidus*. We find that in all previously solved AfAgo structures, its two monomers form substantial dimerization interfaces involving the C-terminal β-sheets. Led by this finding, we have employed various biochemical and biophysical assays, including SEC-MALS, SAXS, single-molecule FRET, and AFM, to show that AfAgo is indeed a homodimer in solution, which is capable of simultaneous interaction with two DNA molecules. This finding underscores the diversity of prokaryotic Agos and broadens the range of currently known Argonaute-nucleic acid interaction mechanisms.

## Introduction

Argonaute (Ago) proteins are found in all three domains of life (bacteria, archaea, and eukaryotes). The best characterized group is eukaryotic Ago (eAgo) proteins. Being the functional core of RNA interference machinery, eAgos are involved in regulation of gene expression, silencing of mobile genome elements, and defense against viruses. From the structural and mechanistic point of view, all eAgos are very similar, as they all use small RNA molecules as guides for sequence-specific recognition of RNA targets, and are monomeric proteins sharing four conserved functional domains, which are organized in a bilobed structure^1,2^. The N-terminal lobe consists of the N-domain that separates guide and target strands, and the PAZ domain responsible for binding the 3′-terminus of guide RNA; the C-terminal lobe consists of the MID domain, which binds the 5′-terminus of guide RNA, and the PIWI domain, an RNase^2–4^. Upon recognition of the RNA target, eAgos may either cleave it employing the catalytic activity of the PIWI domain or, especially eAgo proteins that encode catalytically inactive PIWI domains, recruit partner proteins leading to degradation of the target RNA or repression of its translation^4,5^.

Ago proteins are also identified in 9% of sequenced bacterial and 32% archaeal genomes^6,7^. Unlike eAgos, which exclusively use RNA guides for recognition of RNA targets, different pAgos may use either RNA or DNA guides and/or targets^8,9^, and may also differ in their structural organization. The best understood prokaryotic Ago (pAgo) proteins are the full-length pAgos, which are composed of N, PAZ, MID, and PIWI domains, and thus closely resemble eAgo proteins. It has been demonstrated that full-length pAgos function as prokaryotic antiviral systems, with the PIWI domain performing cleavage of invading nucleic acids^10^. Furthermore, a function beyond immunity—decatenation of circular chromosomes during replication—has also been documented^11^. However, the majority (~ 60%) of identified pAgos are shorter (encode just MID and PIWI domains) and are catalytically inactive due to mutations in the PIWI domain. Though similar artificial truncations of eukaryotic Agos preserve most of the functionality characteristic to full-length proteins^12–15^, the function and mechanism of the naturally-occurring short catalytically inactive pAgos remain unknown^6,7^.

In this work, we focus on the short prokaryotic Argonaute AfAgo encoded by a hyperthermophilic archaeon *Archaeoglobus fulgidus*^6,7^ (UniProtKB O28951). Like other short pAgos, AfAgo contains a MID and a catalytically inactive PIWI domains (albeit sequence analysis suggests that AfAgo MID and PIWI domains are closer to those found in full-length, rather than most short pAgos^6,7^). For over a decade it served as a model system for structural and mechanistic studies of Argonaute-nucleic acids interactions^15–17^. It is also one of the first and one of the best structurally characterized prokaryotic Argonautes, with an apo- and 3 dsDNA/RNA-bound structures currently available^18–21^. However, its biological role, in part due to lack of catalytic activity, remains elusive. Inspection of AfAgo structures revealed that regardless of the crystal form and symmetry, two AfAgo subunits in all cases form substantial dimerization interfaces involving the N-terminal residues and/or β-strands located close to the C-termini. Using various biochemical and biophysical assays, including size exclusion chromatography—multi-angle light scattering (SEC-MALS), single-molecule FRET, small-angle X-ray scattering (SAXS), and atomic force microscopy (AFM), we show that AfAgo indeed forms a homodimer in solution, and is capable of simultaneous interaction with two DNA molecules. This broadens the range of currently known interaction mechanisms involving nucleic acids and Argonaute proteins.

## Materials and methods

### Protein expression

The gene encoding WT AfAgo was amplified from *Archaeoglobus fulgidus* genomic DNA by PCR and cloned into a pETDuet vector, yielding a construct with an N-terminal (His)_6_ tag (N-terminal protein sequence MGSSHHHHHHSQDP (1.63 kDa) followed by 1–427 aa of WT AfAgo sequence). The deletion in the dimerization mutant AfAgoΔ was introduced via overlap extension PCR by using two primer pairs, MZ-385/MZ-875 and MZ-383/MZ-876 (Supplementary table S1) for the N- and C-terminal fragments flanking the region to be deleted, respectively. The two PCR products, possessing a 49 bp overlap, were then used as a template for subsequent PCR with the MZ-383/MZ-385 primers, yielding the full-length fragment, which was then cloned into a pETDuet vector. Both proteins were expressed in *E. coli* strain BL21(DE3). Cells were grown in LB broth in the presence of ampicillin at 37 °C. When A_600_ of the cell culture reached 0.5, the incubation temperature was lowered to 16 °C, 0.1 mM IPTG were added, cells incubated for approx. 16 h at 16 °C and harvested by centrifugation.

### Protein purification

Harvested cells expressing (His)_6_-tagged WT AfAgo or the dimerization mutant AfAgoΔ were disrupted by sonication in buffer A (20 mM Tris–HCl (pH 8.0 at 25 °C), 500 mM NaCl, 5 mM 2-mercaptoethanol) with 2 mM phenylmethylsulfonyl fluoride, incubated for 20 min at 50 °C and cell debris was removed by centrifugation at 48,400*g* for 1 h. The supernatant was loaded onto a HiTrap chelating HP column charged with Ni^2+^ (GE Healthcare) and eluted with a linear gradient (15–500 mM) of imidazole in buffer A. The fractions containing protein were pooled, diluted to 0.2 M of NaCl with a buffer containing 20 mM Tris–HCl (pH 8.0 at 25 °C), 10% (v/v) glycerol, 5 mM 2-mercaptoethanol and incubated for 1 h at 37 °C with 1 mM EDTA (ethylenediaminetetraacetic acid) and RNase A/T1 (ThermoFisher Scientific) (1:100). Next, the protein solution was centrifuged at 48,400*g* for 30 min, the supernatant containing RNA-free AfAgo was loaded onto a HiTrap Heparin HP column (GE Healthcare) and eluted using a 0.2–1.0 M NaCl gradient. Finally, the protein was run through the HiLoad 16/600 Superdex 200 column (GE Healthcare) in buffer A supplemented with NaCl to 1 M and dialyzed against 20 mM Tris–HCl (pH 8.0 at 25 °C), 500 mM NaCl, 50% (v/v) glycerol.

### SEC-MALS

The samples of WT AfAgo or AfAgoΔ in the absence of nucleic acids (final protein concentration 1.0 mg/mL or 20 µM in terms of monomer, injected volume 2.0 mL) were separated using a HiLoad 16/60 Superdex 200 prep grade column (GE Healthcare). The column was equilibrated with a buffer containing 100 mM Tris–HCl pH 8.0, 1000 mM NaCl and 0.04% (w/v) NaN_3_, the flow rate was 0.5 mL/min. Samples of AfAgo with 5′-phosphorylated MZ-1289 DNA (final concentration 5 µM protein monomer and 5 µM DNA oligoduplex) were separated on a Superdex 200 Increase 10/300 GL column equilibrated with a buffer containing 15 mM Tris–HCl pH 8.0, 150 mM NaCl, 5 mM MgCl_2_, 0.5 mM 1,4-dithiothreitol and 0.04% (w/v) NaN_3_, flow rate was 0.4 mL/min. The light scattering signals were monitored on a miniDawn TREOS II detector, concentrations of apo-protein samples were measured using an Optilab T-rEX refractive index detector (Wyatt Technologies), concentrations of protein–DNA complexes were measured using both refractive index and UV absorption (Waters 2487 UV detector) readings. Data were analyzed in Astra software (Wyatt Technologies) using dn/dc values of 0.185 g/mL and 0.170 g/mL for protein and DNA, respectively. Scattering data of protein–DNA complexes was analyzed using the “protein conjugate” method in Astra; the required DNA and apo-protein UV extinction coefficients were determined experimentally using the “UV extinction from RI peak” method and the DNA-only and apo-protein samples.

### Small-angle X-ray scattering

Small-angle scattering data of WT AfAgo and monomeric mutant were collected at the P12 EMBL beamline on the PETRA III ring of the DESY synchrotron in Hamburg, Germany^22^. Details of data collection and principal structural parameters are presented in Supplementary table S2 and Supplementary figure S1. Protein complexes with DNA (MZ-1289) were transferred to sample buffer (20 mM Tris–HCl (pH 7.5 at 25 °C), 5 mM MgCl_2_, 150 mM NaCl and 2 mM 1,4-dithiothreitol) using Illustra NAP columns (GE Healthcare).Dimeric AfAgo complex with MZ-1289 was analyzed by SEC-SAXS with an Agilent FPLC system. The AfAgo + MZ-1289 was concentrated to 175 µM and loaded on the column Superdex 200 Increase 10/300 (GE Healthcare) equilibrated with the sample buffer. Frames collected during the complete SEC run (flow rate of 0.5 ml/min, 3000 frames) were analyzed with CHROMIXS^23^, frames corresponding to the peak were averaged and processed. Ab initio shape determination was carried out by generating 20 independent DAMMIF^24^ models using parameterized scattering curves created by GNOM^25^ under P2 symmetry restraints. Models were clustered by DAMCLUST^26^ and models forming a cluster were averaged by DAMAVER^27^ and used as a starting model for an additional run of DAMMIN^28^.

SAXS measurements performed with a range of AfAgoΔ concentrations (1–10 mg/ml) showed significant protein aggregation. The pseudo-chain dummy residues models of the complex generated by GASBOR^29^ were superimposed with crystallographic dimers of AfAgo as well as with the monomeric AfAgo-DNA complex using SUPCOMB^30^ applying step-wise shift (5 Å) along the principal axis of the model as described in^31^.

**Figure 1:**
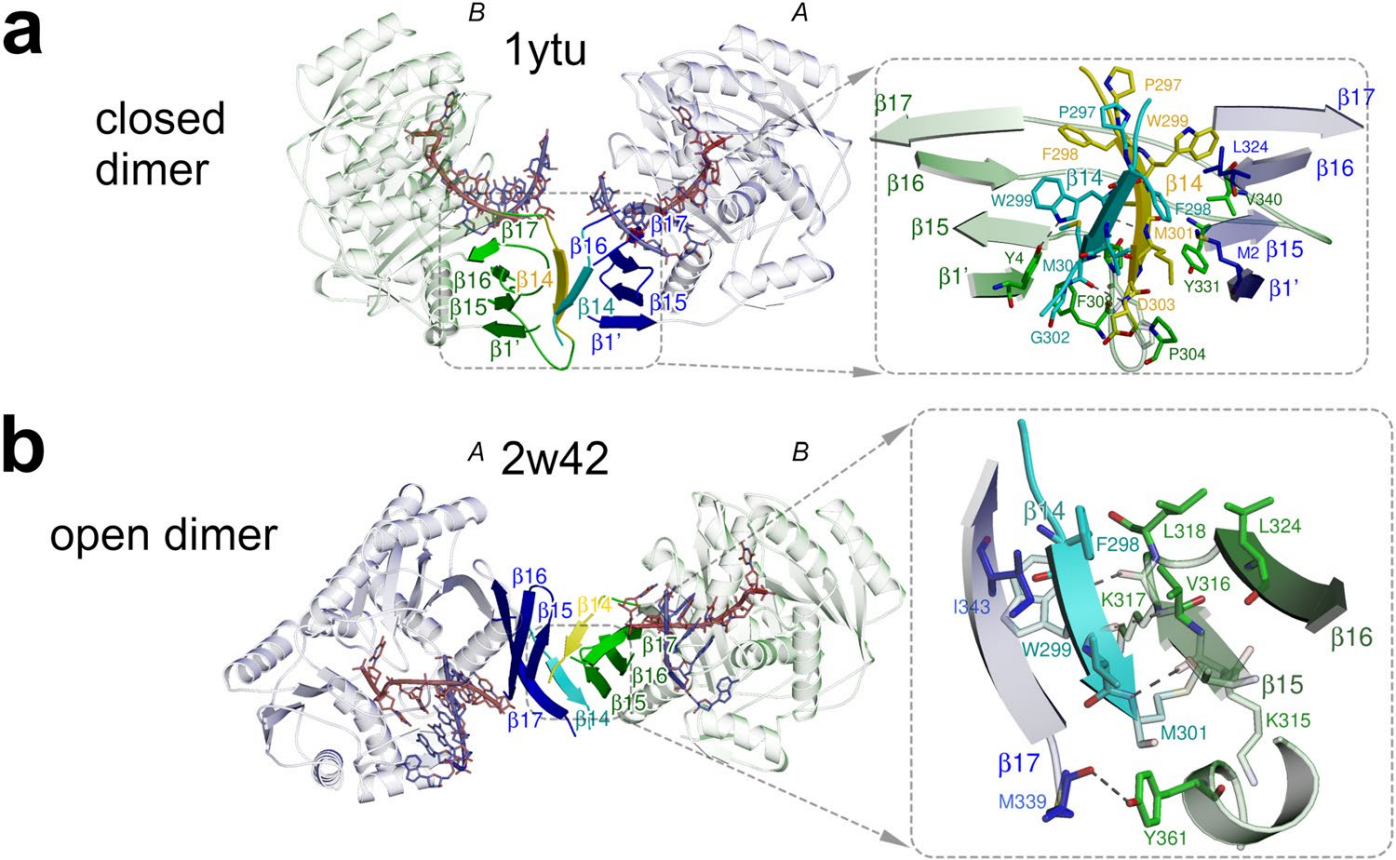
Dimerization of AfAgo. (**a**,**b**) Protein subunits are coloured blue (protein chain *A*) and green (protein chain *B*). The interface-forming secondary structure elements are highlighted and numbered according to the PDB ID 2w42 assignment made by PDBsum^52^. The “guide” DNA/RNA strands bound by AfAgo are coloured red, “target” strands—blue. Residues 296–303 deleted in AfAgoΔ are coloured cyan and yellow. Hydrogen bonds are shown as dashed lines. (**a**) AfAgo complex with dsRNA (PDB ID 1ytu, both protein chains as present in the asymmetric unit), the “closed” dimer^19^. (**b**), AfAgo complex with dsDNA (PDB ID 2w42)^21^)—the “open” dimer. β-strands from both subunits assemble into a closed β-barrel structure, with intersubunit interface formed by β14 and β15 strands of neighbouring subunits.

The SAXS data was compared to crystal structures using CRYSOL^32^ (Fig. 1). Particle volume and M_w_ estimations were performed using several methods (Supplementary table S3 and references therein).

### DNA fragments

DNA fragments were assembled and prepared as depicted in Supplementary Fig. S2. All full-length DNA fragments were subsequently purified from an agarose gel using a runVIEW system (Cleaver Scientific, UK), precipitated with sodium acetate/isopropanol, washed with 75% (v/v) ethanol, and resuspended in water.

### AFM sample preparation and imaging

DNA–protein complexes were formed by incubating the DNA fragment (5 nM) with WT AfAgo or AfAgoΔ (concentration in terms of monomer 50 nM) for 5 min at room temperature in the Binding Buffer HEPES (33 mM HEPES (pH 7.8 at 25 °C), 66 mM CH_3_COOK, 5 mM (CH_3_COO)_2_ Mg) in a total volume of 50 μl. Next, the protein–DNA complexes were cross-linked with 2.5% (w/v) glutaraldehyde for 20 min. Glutaraldehyde was then quenched with an excess of the Tris buffer (33 mM Tris–acetate (pH 7.8 at 25 °C), 66 mM CH_3_COOK, 5 mM (CH_3_COO)_2_ Mg). The resultant reaction solution after tenfold dilution with Tris buffer was deposited onto modified mica at room temperature as described below.

Freshly cleaved muscovite mica (grade IV, SPI supplies Inc., USA) was incubated in a mixture of 1-(3-aminopropyl)-silatrane (APS) solution for 30 min to prepare functionalized APS-mica, as described previously for the preparation of protein–DNA complexes^33^. 50 μl of DNA–protein complex solution was deposited on APS-mica for 5 min. After incubation the mica surface was immersed in deionized water for 5 min, flushed with excess water, and then dried under a flow of nitrogen. The images were acquired in the air with “DimensionIcon” (Bruker, Santa Barbara, CA) microscope system in tapping mode. Probes with nominal spring constants of ~ 5 or 40 N/m were used. Typically, the images were collected at a speed of 0.6 Hz and a resolution of 1024 × 1024 pixels, scan size 2 µm × 2 µm.

### Single-molecule fluorescence microscopy

The overall idea of fluorescence burst data acquisition of single diffusing molecules in alternating laser excitation (ALEX) mode was based on^34^. The principal opto-mechanical layout of the experiment is shown in Supplementary Fig. S3 and described in Supplementary Data.

The measurement of single surface-immobilized molecules with the excitation in the total internal reflection mode (TIR) was performed on the same setup exploiting its alternative functionality as described previously^35^. Briefly, the objective was 100 × 1.4 Oil Plan Apo VC (Nikon), the fluorescence signal was split by T640lpxr-UF2 dichroic mirror (Chroma) and the different spectral channels were projected on the same EMCCD (DU-897ECS0-UVB, Andor).

### Sample cell preparation for single-molecule measurements

FRET bursts measurements were performed in a chambered coverglass well (155411, Nunc Lab-Tek, Thermo Scientific). The reaction volume was 200 µl. The reaction buffer (RB) was 33 mM Tris–acetate (pH 7.9 at 20 °C), 66 mM CH_3_COOK, 5 mM (CH_3_COO)_2_ Mg, and 0.1 mg/ml bovine serum albumin. The DNA concentration was 17–50 pM. Measurements at different protein concentrations were carried out by adding to the reaction small volumes of protein diluted in RB in “Protein LoBind” 1.5 mL tubes (Eppendorf). No oxygen-scavenging or triplet-quenching additives were used.

Measurements of surface-immobilized DNA fragments were performed in a flow cell assembled from a six-channel Sticky-Slide VI 0.4 (Ibidi) and a coverslip functionalized with polyethylene glycol derivatives as described in detail in^35^. The flow cell was incubated with 5 µg/ml of Neutravidin (Molecular probes) in RB for 2 min, washed with RB, incubated with 5 pM DNA in RB until the density of the surface-immobilized DNA fragments appeared to be appropriate, and washed with RB. For the measurement, a 20 nM solution of AfAgo in RB supplemented with 1% (w/v) glucose (TCI Europe), 2.5 mM Trolox (Sigma-Aldrich), and 15 U/ml glucose oxidase (Sigma-Aldrich) was injected into the cell. Trolox was treated with UV light for 20 min according to Cordes et al.^36^. Single-molecule data analysis was performed as described in Supplementary Data.

## Results

### AfAgo is a homodimer in the available X-ray structures

AfAgo is a 427 amino acid (aa) 49.2 kDa prokaryotic Argonaute protein found in the hyperthermophilic archaeon *Archaeoglobus fulgidus*. To date, four AfAgo structures, both of the apo-form and bound to DNA and RNA duplexes were solved^18–21^. AfAgo monomer is composed of two major domains, the N-terminal MID (residues 38–167), and the C-terminal PIWI (residues 168–427)^18^. The MID domain specifically binds the 5′-phosphorylated end of the presumed guide DNA/RNA strand, and also makes contacts to the complementary target DNA/RNA strand^19–21^. The PIWI domain makes contacts to both guide and target DNA/RNA strands but is catalytically inactive due to mutations in the RNase H-like catalytic centre. Inspection of available AfAgo structures (Supplementary table S4) revealed that in all structures known so far, AfAgo subunits form substantial homodimerization interfaces, which were not further scrutinized by authors of the corresponding structural studies. The dimerization interface in the AfAgo-dsRNA structure (PDB ID 1ytu) is asymmetric and primarily involves the C-terminal β-strands (residues 296–303) from both subunits present in the asymmetric unit that together form a parallel β-sheet, and the N-terminal residues from one of the subunits (Fig. 1a). The dimer formed in this case is compact (henceforth, a “closed” dimer). In contrast, dimerization interfaces in three other cases (PDB IDs 1w9h, 2bgg, and 2w42) are nearly symmetrical with respect to the secondary structure elements involved (albeit in PDB IDs 2bgg and 2w42 they belong to different protein chains present in the asymmetric unit): the C-terminal β-strands form 8-strand β-barrels, with the sheets from different subunits interacting via strands β14 (residues 297–302) and β15 (residues 314–318, Fig. 1b). The resultant dimers are less compact (henceforth, “open” dimers).

The solvent-accessible surface areas buried at the dimerization interfaces in both “open” and “closed” dimers are classified as “significant” by the PISA server (https://www.ebi.ac.uk/pdbe/pisa/pistart.html^37^; Supplementary table S4). This observation prompted us to test the oligomeric state, the possible dimerization mode, and the mechanism of nucleic acid binding of AfAgo in solution using various biochemical and biophysical techniques. For that purpose, we used two variants of AfAgo: the full-length wild-type protein (henceforth, WT AfAgo), and a dimerization mutant AfAgo lacking the 296–303 amino acid residues responsible for the majority of dimerization contacts in both the “closed” and “open” homodimers (henceforth, AfAgoΔ, Fig. 1a,b). Both proteins were successfully purified as described in “Materials and methods”.

### SEC-MALS analysis

**Figure 2:**
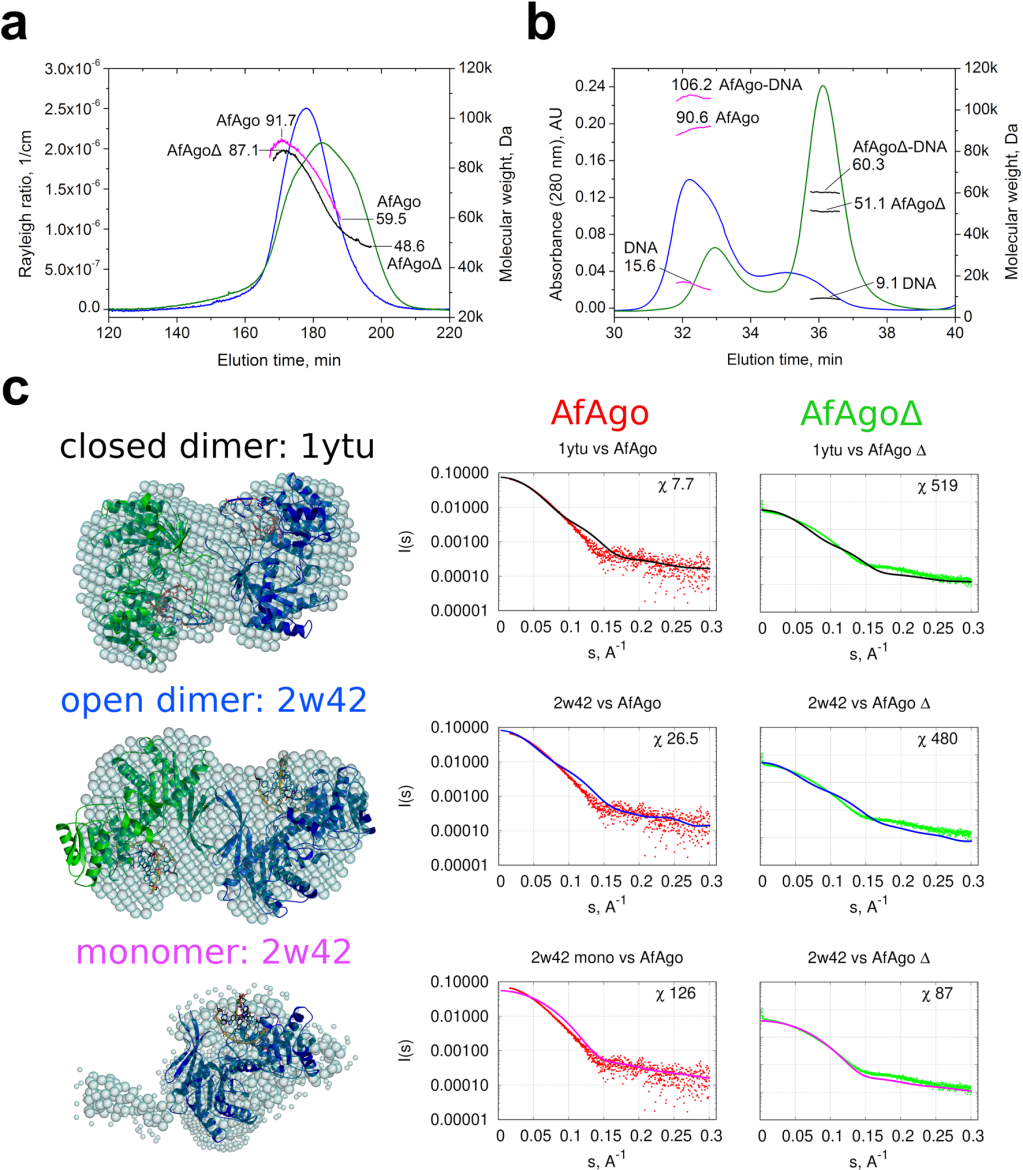
SEC-MALS and SAXS analysis of apo-AfAgo and AfAgo-DNA complexes. (**a**) SEC-MALS analysis of WT AfAgo and dimerization mutant AfAgoΔ unbound to nucleic acids. The light scattering data (blue for WT AfAgo, green for AfAgoΔ) is shown along with the calculated M_w_ values (magenta for WT, black for mutant). The highest and lowest M_w_ values calculated for each protein are indicated. Theoretical M_w_ of WT AfAgo monomer is 50.8 kDa, theoretical M_w_ of AfAgoΔ monomer is 49.9 kDa. (**b**) SEC-MALS analysis of AfAgo-DNA and AfAgoΔ-DNA complexes. The UV absorption data of AfAgo-DNA (blue) and AfAgoΔ-DNA (green) is shown along with the M_w_ values of full complexes, the protein component, and the DNA component (magenta for AfAgo-DNA sample and black for AfAgoΔ-DNA sample, respectively). The theoretical M_w_ of a 2:2 WT AfAgo:DNA complex is 119 kDa (2 × 50.8 + 2 × 8.7 kDa), theoretical M_w_ of a 1:1 AfAgoΔ-DNA complex is 58.6 kDa (49.9 + 8.7 kDa). (**c**) SAXS data of WT AfAgo complex with MZ-1289 DNA (red points) and the dimerization mutant AfAgoΔ with MZ-1289 DNA (green points) are compared with the scattering curves generated from the “closed” dimer with dsRNA (PDB ID: 1ytu, black curves), “open” dimer (PDB ID: 2w42, blue curves) and AfAgo-DNA complex (PDB ID: 2w42, magenta curves) by CRYSOL. Corresponding AfAgo structures are shown in the second column superimposed with the dummy atom models generated using the SAXS data of the AfAgo complex with MZ-1289 oligoduplex.

First, we tested the oligomeric state of WT AfAgo and AfAgoΔ proteins using size exclusion chromatography—multi-angle light scattering (SEC-MALS). We find that WT AfAgo elutes from the SEC column as a polydisperse peak (Fig. 2a), with the M_w_ values ranging from 91.7 kDa at the left-hand side of the peak (close to the theoretical M_w_ of WT AfAgo homodimer, 101.6 kDa) to 59.5 kDa at the right-hand side (still considerably higher than M_w_ of a WT AfAgo monomer, 50.8 kDa). AfAgoΔ formed a far broader irregular peak, covering M_w_ values from 87 kDa (close to M_w_ of AfAgoΔ dimer, 99.8 kDa) to approx. 49 kDa (close to AfAgoΔ monomer, 49.9 kDa). We conclude that WT AfAgo indeed forms homodimers, which under our experimental conditions (~ 10 µM concentration in the sample, ~ 1.5 µM concentration on the column) are relatively unstable and partially dissociate into monomers. Deletion of the 296–303 residues in the AfAgoΔ protein further decreased the stability of the dimer, in line with their proposed role in dimerization (Fig. 1). Intriguingly, the differences between WT AfAgo and AfAgoΔ oligomeric states were more pronounced in their DNA-bound forms (WT AfAgo-DNA and AfAgoΔ-DNA, respectively): the majority of WT AfAgo-DNA eluted as a 2:2 protein:DNA complex, while the major peak of AfAgoΔ-DNA matched a 1:1 protein:DNA complex (Fig. 2b).

### SAXS measurements

To characterize the conformation of AfAgo in solution, we have performed small-angle X-ray scattering (SAXS) measurements using WT AfAgo-DNA and AfAgoΔ-DNA complexes. Two types of data analysis were performed: (i) the ab initio shapes of the complexes in solution were calculated and superimposed with the X-ray AfAgo structures, and (ii) the theoretical scattering data was calculated for the crystallized DNA-bound AfAgo monomer, “open” (PDB ID: 2w42 and 1w9h) and “closed” (PDB ID: 1ytu) dimers, and compared to experimental SAXS scattering data of AfAgo-DNA and AfAgoΔ-DNA (Fig. 2). The “closed” AfAgo dimer fits the AfAgo-DNA SAXS data better than the “open” dimer, as judged from the real space fit and the χ^2^ (Fig. 2c) parameters, implying that in solution WT AfAgo predominantly forms a “closed” dimer. As expected, the AfAgo monomer gave the best fit to the AfAgoΔ-DNA SAXS data (Fig. 2c, right column). The SAXS molecular weights calculated for WT AfAgo-DNA (between 94.2 and 106.9 kDa, Supplementary table S3) agreed with the expected mass of the dimer complexed with dsDNA (119 kDa). The SAXS M_w_ for the AfAgoΔ-DNA (between 55.4 and 67.9 kDa) confirmed its monomeric state.

### Direct visualization of AfAgo-induced DNA loops by AFM

We have observed the dimeric state of WT AfAgo in X-ray structures, SEC-MALS, and SAXS measurements, i.e. techniques that all require relatively high (micromolar and higher) protein and DNA concentrations. This raises a question if AfAgo dimerisation and the ability to simultaneously interact with two nucleic acid molecules are relevant in solution at far lower protein and DNA concentrations. To address this question, we have examined AfAgo interactions with long DNA molecules by AFM and single-molecule FRET.

**Figure 3:**
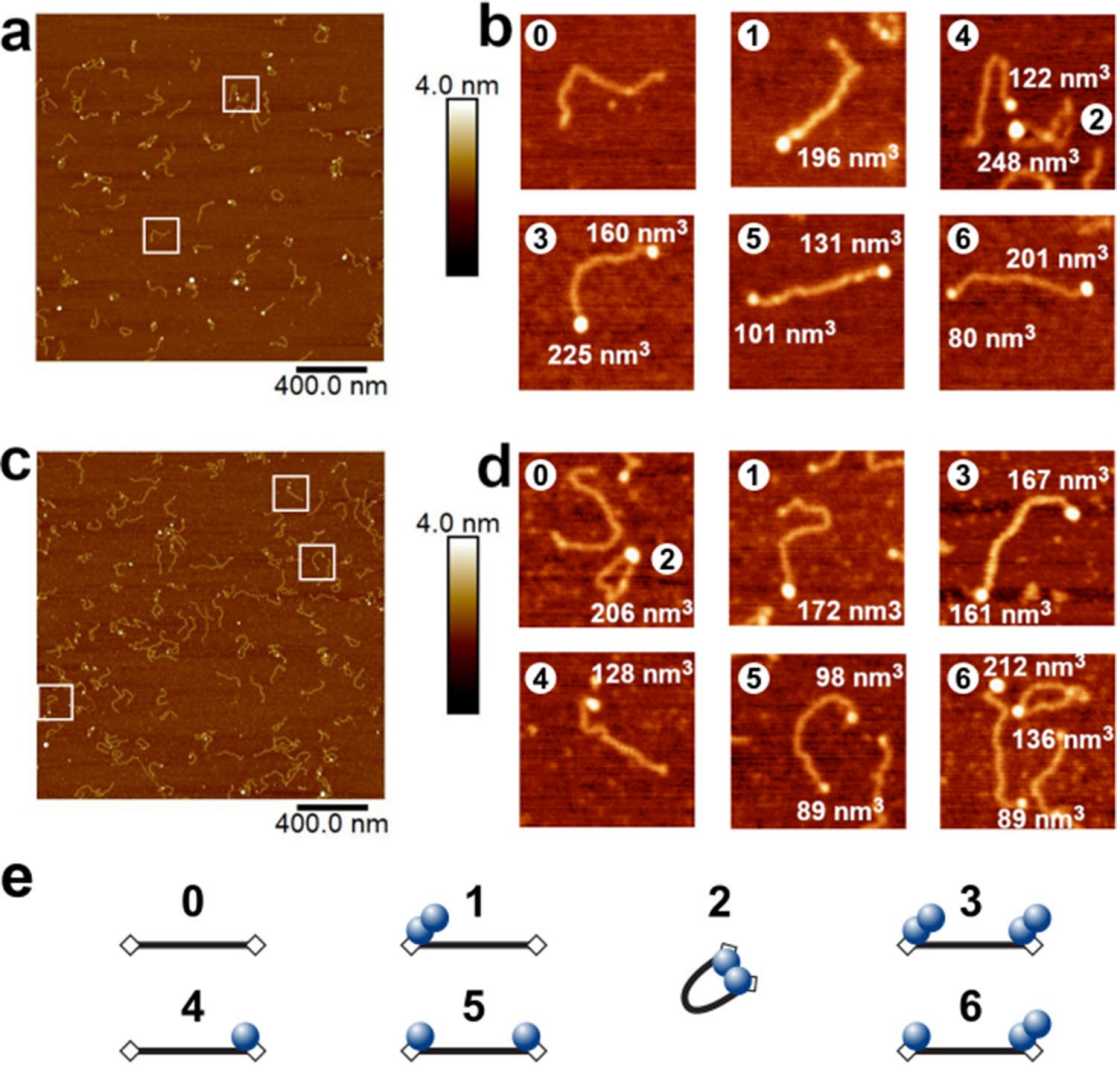
Visualization of AfAgo-induced DNA loops by AFM. Representative AFM images and fourfold enlarged views of WT AfAgo-DNA (**a**,**b**) and AfAgoΔ-DNA (**c**,**d**) complexes adsorbed to APS-mica acquired in the air are shown, along with calculated protein volumes. Area of each image in the (**a**,**c**) is 4 µm^2^, scale bar is 400 nm, the Z range is 4 nm; the Z range of images in (**b**,**d**) is 3.0 nm. Regions marked by white squares in (**a**,**c**) indicate several of the observed protein–DNA complexes enlarged in (**b**,**d**). Based on protein volumes, we assign AfAgo-DNA complexes shown in (**b**,**d**) to different protein–DNA stoichiometries and arrangements (numbered from 0 to 6) that are schematically depicted in (**e**): “0”—naked DNA; “1”—AfAgo dimer bound to one DNA end; “2”—AfAgo dimer forming a DNA loop; “3”—two AfAgo dimers on different DNA ends; “4”—an AfAgo monomer on one DNA end; “5”—two AfAgo monomers on different DNA ends; “6”—a monomer and a dimer on different DNA ends.

For direct visualization of protein–DNA complexes, AfAgo and DNA (a 585 bp blunt-end PCR fragment with 5′-phosphorylated termini) were deposited on APS-mica and imaged using tapping AFM. A typical AFM image of AfAgo-DNA complexes is shown in Fig. 3. Several types of protein–DNA complexes, shown as enlarged insets in Fig. 3, were observed: (i) linear DNA with a protein molecule bound to one DNA end; (ii) linear DNA with protein molecules bound to both DNA ends; (iii) ring-shaped (looped) DNA. Other species, including naked DNA, or more complex structures, involving, e.g., protein bound to two DNA fragments, were also observed but were not quantified. Analysis of protein volumes in the AfAgo-DNA complexes revealed a broad distribution of sizes, ranging from approx. 60 nm^3^ (lower value than expected for an AfAgo monomer, approx. 100 nm^3^), to above 270 nm^3^ (higher value than expected for an AfAgo dimer, approx. 200 nm^3^), albeit the average particle size was considerably smaller in the case of the AfAgoΔ mutant (Supplementary Fig. S4). DNA length in the analysed protein–DNA complexes also showed considerable variation, with the average values close to 195 nm, the theoretical length of 585 bp DNA. However, we did not observe any correlation between calculated DNA length and protein volumes in the complexes, confirming that DNA made no systematic contribution to the measured protein sizes (Supplementary Fig. S5).

Notably, we find that the relative distribution of different complexes varied dramatically for WT AfAgo and the dimerization mutant AfAgoΔ (Table 1). The ring-shaped DNA–protein complexes is the dominant species observed with WT AfAgo (51% or 95 out of 187 complexes, 47 of them containing a dimeric protein). A minor fraction of DNA molecules had either protein bound to one end (35%, 66 out of 187 complexes, 33 of them monomers and 22 dimers) or to both ends (13%, 26 out of 187, 6 of them two monomers, 4 of them two dimers, 13 one monomer and one dimer). In the case of AfAgoΔ, the majority of complexes had protein bound either to both DNA ends (34%, or 58 out of 169, 20 of them—two monomers, 8—two dimers, 25—one monomer and one dimer, Table 1) or to one end (40%, or 58 out of 169, 38 of them monomers and 24 dimers). A much smaller fraction (26%, or 44 out of 187) were ring-shaped structures. We assume that ring-shaped DNA molecules are primarily formed by dimeric WT AfAgo bound to both termini of the DNA fragment, in a similar manner as observed in the X-ray structures. A prominent decrease in ring-shaped DNA in the AfAgoΔ samples is consistent with its impaired dimerization. The remaining looped complexes are likely formed due to the residual ability of AfAgoΔ to form dimers, though we cannot exclude inadvertent cross-linking of DNA-bound AfAgoΔ monomers with glutaraldehyde during sample preparation (see “Materials and methods” for details). Cross-linking may also account for the presence of higher order AfAgo oligomers observed by AFM (Table 1).

**Table 1:** AfAgo-DNA complexes observed by AFM (see Supplementary Fig. S4).

Protein	Complex		
	DNA loops, %	Linear	
		Protein bound to one end, %	Protein bound to both ends, %
*WT AfAgo*	51% (n = 95)	35% (n = 66)	13% (n = 26)
	Monomer (n = 20)^a^ Dimer (n = 47) Other (n = 28)	Monomer (n = 33) Dimer (n = 22) Other (n = 11)	Monomer–monomer (n = 6) Dimer-dimer (n = 4) Monomer–dimer (n = 13) Other (n = 3)
*AfAgoΔ*	26% (n = 44)	40% (n = 67)	34% (n = 58)
	Monomer (n = 24) Dimer (n = 12) Other (n = 8)	Monomer (n = 38) Dimer (n = 24) Other (n = 5)	Monomer–monomer (n = 20) Dimer-dimer (n = 8) Monomer–dimer (n = 25) Other (n = 5)

^a^The calculated volume of WT AfAgo (50.8 kDa) and AfAgoΔ (49.9 kDa) proteins is *approx.* 100 nm^3^. Thus, the measured protein volume data from AFM images was divided into three populations by their theoretical volume: *monomer* (< 150 nm^3^), *dimer* (150–250 nm^3^), and *other* (> 250 nm^3^). For details *see* Supplementary Data and Figure S4.

### WT AfAgo induces DNA loops in solution

**Figure 4:**
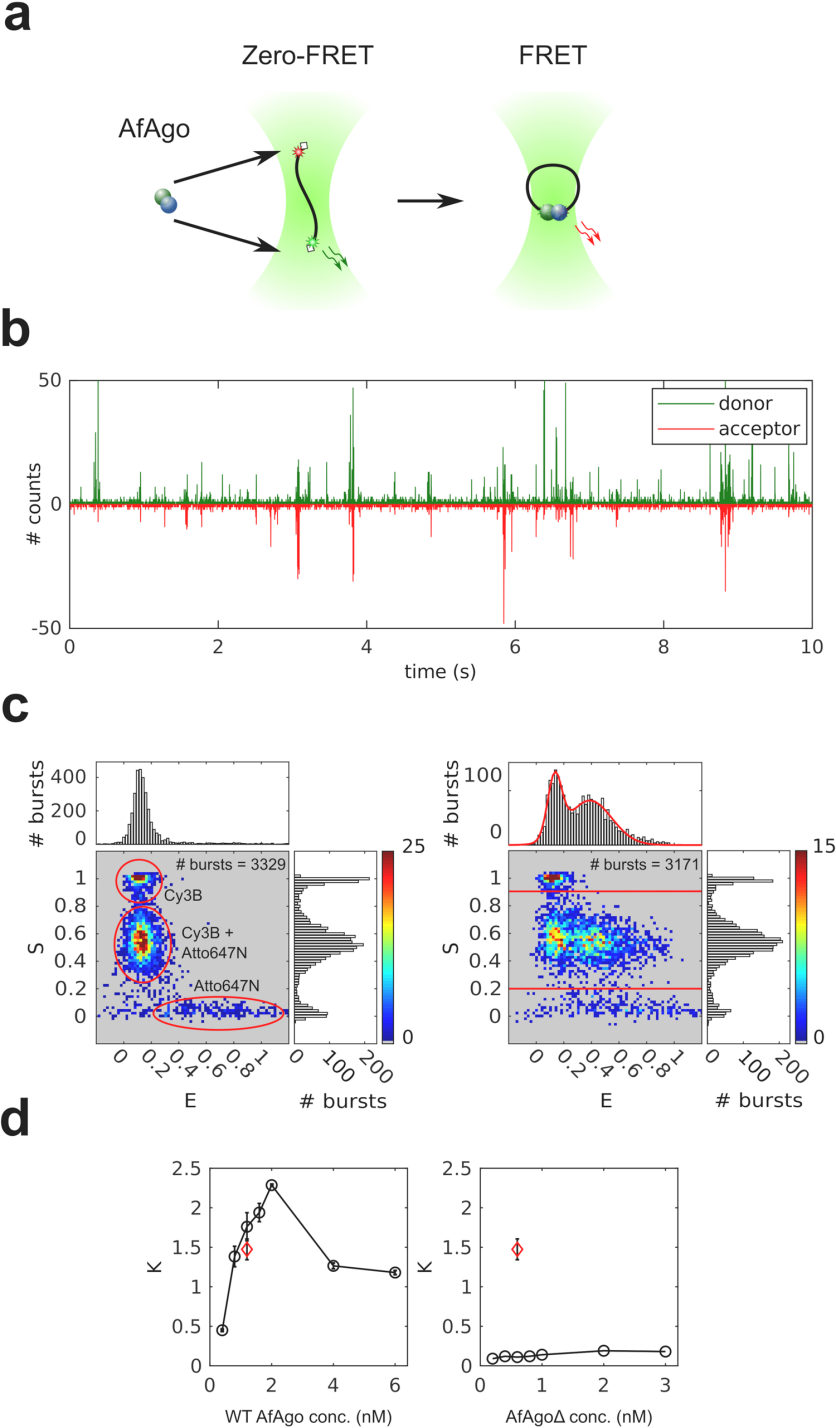
Single-molecule studies of AfAgo-DNA interactions in solution. (**a**) A schematic overview of the single-molecule assay. Left, free DNA; right, WT AfAgo-DNA (blue and green circles) complex in a looped state. (**b**) Fluorescence intensity trace with 1 ms time bin of 25 pM DNA with 2 nM AfAgo. Red: inverted acceptor fluorescence upon donor excitation, green: donor fluorescence upon donor excitation. (**c**) Left—E-S histogram of DNA alone. The top and side axes contain, respectively, one-dimensional E (proximity ratio) and S (donor/acceptor stoichiometry) histograms of all bursts. Denoted are areas representing donor-only DNA, acceptor-only DNA, and dual-labelled DNA. Right—E-S histogram of DNA with 2 nM AfAgo. The one-dimensional E histogram on top is derived from bursts with S = 0.2–0.9, designated by horizontal lines in the E-S histogram. The red curve is a two-Gaussian fit to the data that gave positions of the Gaussian maxima on the E-axis (0.13 ± 0.01 and 0.39 ± 0.02). (**d**) Left—dependence of the ratio of looped and unlooped DNA molecules (parameter K) on WT AfAgo concentration (open circles). Right—the dependence of K on the AfAgoΔ concentration (open circles). Red diamonds in both graphs represent the competition experiment performed with 1.2 nM WT AfAgo and 0.6 nM AfAgoΔ. All data points are average values of three measurements ± 1 standard deviation.

To further characterize AfAgo-DNA interactions at nanomolar concentrations, we have examined AfAgo-DNA interactions using single-molecule Förster resonance energy transfer (smFRET). If AfAgo homodimer simultaneously interacts with two ends of the same DNA molecule, the induced DNA loops can be monitored as a change in FRET efficiency between dyes tethered close to DNA ends (Fig. 4a). Utilization of a single dual-labelled DNA substrate (rather than two short DNA duplexes carrying different fluorescent labels) increases the probability of AfAgo interaction with both DNA ends at low reactant concentrations required for the single-molecule setup.

We have designed a 569 bp DNA construct, which was labelled with a pair of FRET fluorophores, Cy3B and Atto647N, each attached to thymine bases 3 nt away from the respective DNA termini via C6 linkers (Supplementary Fig. S2). Positions of the FRET labels were selected such that upon binding of both DNA ends by an AfAgo dimer, the distance between the label attachment sites (irrespective of the AfAgo dimerization mode), is favourable for FRET (Supplementary Fig. S6), and that attached labels do not interfere with AfAgo binding to DNA (Supplementary Fig. S7).

AfAgo interaction with the DNA fragment was monitored by analyzing the fluorescence bursts of single diffusing DNA fragments (Fig. 4b). As described in Supplementary Materials and Methods, for each DNA molecule we have calculated the stoichiometry parameter S, which is close to 0.5 for DNA molecules labelled with both fluorophores, approx. 0 for the acceptor-only DNA, and close to 1.0 for the donor-only DNA, and the proximity ratio E, which is expected to be higher for looped DNA molecules with the FRET dyes brought into close proximity than for unlooped DNA molecules.

The E-S histogram of DNA alone (Fig. 4c, left) exhibits a prominent population with low E and intermediate S values, which corresponds to dual-labelled unlooped (zero-FRET) DNA molecules. The two minor populations observed in the histogram correspond to donor-only (low E/high S) and acceptor-only (high E/low S) DNA fragments.

The E-S histogram of DNA in the presence of WT AfAgo exhibits an additional population (intermediate S and intermediate E, Fig. 4c, right), which presumably represents DNA molecules looped by WT AfAgo. The fraction of looped and unlooped DNA molecules was quantified by fitting a sum of two Gaussian functions to the 2D histogram of E values of dual-labelled molecules (Fig. 4c, right), and finding the areas under the Gaussian with a relatively high E centre (representing looped DNA) and a Gaussian with a near-zero E centre (representing unlooped DNA). DNA looping efficiency K was then defined as the ratio of the two areas.

We have measured the ratio K at different WT AfAgo concentrations (Fig. 4d). It increased monotonously with increasing WT AfAgo concentration until it reached the maximum value of 2.5 (corresponds to about 70% of looped DNA molecules) at 2 nM WT but decreased as the protein concentration was increased further.

A similar set of single-molecule experiments was performed with the dimerization mutant AfAgoΔ. As shown in Fig. 4d, the ratio K at all AfAgoΔ concentrations tested was close to zero, indicating that AfAgoΔ was unable to induce DNA loops. Lack of DNA looping was not due to impaired DNA binding, as shown by electrophoretic mobility shift assay (Supplementary Fig. S7). Moreover, AfAgoΔ competes with WT AfAgo for DNA ends, as the K value observed in a competition experiment performed with equal concentrations of WT AfAgo dimer and AfAgoΔ monomer was considerably lower than in an experiment with WT AfAgo alone (Fig. 4d). Taken together, efficient DNA looping observed with WT AfAgo, and impaired looping by the dimerization interface mutant AfAgoΔ, provide further support for the ability of WT AfAgo dimer to simultaneously bind two DNA ends in solution.

### Dynamics of WT AfAgo-induced DNA loops

**Figure 5:**
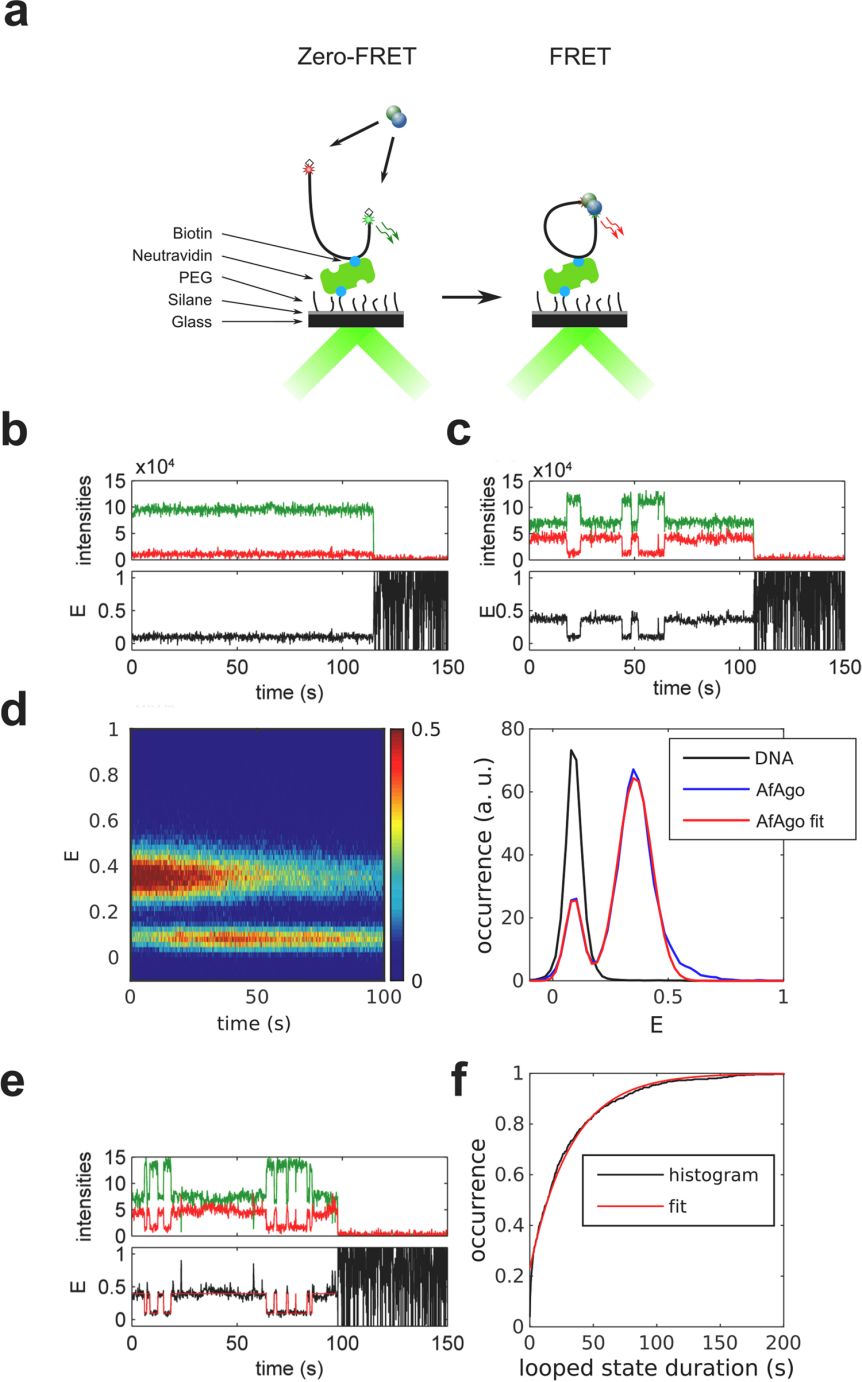
Dynamics of WT AfAgo-induced DNA loops. (**a**) A schematic overview of the single-molecule assay using TIRF microscopy. (**b**,**c**) Trajectories of donor (green) and acceptor (red) intensity and corresponding proximity ratio, E, of individual DNA fragments without (**b**) and with 20 nM WT AfAgo (**c**). (**d**) Left—an image of 287 pooled time traces of the proximity ratio, E, from the measurement with 20 nM of WT AfAgo. The image is normalized to the maximum image intensity. Right—a section of the image in the left integrated over the first 10 s shown with the two-Gaussian fit. The positions of the Gaussian maxima are 0.09 ± 0.01 and 0.36 ± 0.01. For comparison, a trace- and time-averaged section from the measurement of 227 traces on bare DNA is shown. (**e**) An example of trajectories of donor (green) and acceptor (red) intensity and corresponding proximity ratio, E, with HMM idealization of an individual DNA fragment with 20 nM WT AfAgo. (**f**) Cumulative histogram of the looped state durations from 287 E traces with a single-exponential fit with the exponential factor of 33 ± 1 s.

To explore the dynamics of the WT AfAgo-induced DNA looping events, we have used total internal reflection fluorescence (TIRF) microscopy to perform single-molecule FRET experiments on surface-immobilized DNA (Fig. 5). For that purpose, we used a DNA fragment that was essentially identical to the one used for single-molecule studies in solution, except that it carried a biotin 386 bp away from the donor end for surface immobilization (Supplementary Fig. S2). After verifying that WT AfAgo induces loops on this substrate in solution (Supplementary Fig. S8b), we immobilized the biotinylated DNA on a surface and then recorded fluorescence movies in the absence or in the presence of WT AfAgo (Supplementary Fig. S8a). From each frame of the movie we have extracted donor and acceptor intensities for individual DNA fragments, selected trajectories with anti-correlated changes of the donor and acceptor intensities (indicating the occurrence of FRET), and calculated the time courses of the proximity ratio, E. An example of such a trajectory is presented in Fig. 5c. In a control with no AfAgo, we could find no DNA fragments exhibiting FRET (Fig. 5b).

The single-molecule population and time-averaged E values exhibit two peaks with maxima at 0.09 and 0.36, corresponding to the unlooped and looped DNA molecules, respectively (Fig. 5d, right). These E values are also in good agreement with the E values obtained from the measurement in solution (Fig. 4c).

A superficial inspection of E trajectories of individual DNA fragments revealed that their looping dynamics are rather diverse. There exist trajectories with the looped state lasting the whole measurement, whereas other trajectories are more dynamic with a number of transitions between the looped and unlooped states (Fig. 5c, Supplementary Fig. S8c,d). The looped E state also exhibits more subtle dynamics (Supplementary Fig. S8d), which we attribute to the conformational flexibility of AfAgo at the dimerization interface.

To quantify the looped state duration, we first idealized the E trajectories using HMM with a two-state model in QuB software^38^ (Supplementary Materials and methods) (Fig. 5e), and then built the cumulative histogram of the looped state durations (Fig. 5f). The trajectory edge dwells were not omittedto preserve the information on the occurrence of states lasting during the whole trajectory. The exponential factor of a single-exponential fit to the cumulative histogram was 33 ± 1 s. However, the maximum recorded looped state duration is limited by the duration of our measurement (200 s) and the duration of the fluorescent state of the fluorophores before photobleaching. The value of the exponential factor thus sets the lower limit for the looped state duration.

## Discussion

All characterized long Argonaute proteins interact with their RNA and/or DNA targets as monomers, binding a single copy of each guide and target nucleic acids. Surprisingly, we demonstrate here that AfAgo, a prokaryotic Argonaute from the hyperthermophilic archaeon *Archaeoglobus fulgidus*, follows a different mechanism, which involves homodimerization and simultaneous interaction with two guide-target nucleic acid duplexes.

First, we show that AfAgo is a homodimer in all previously solved X-ray structures, including apo-protein, and complexes with RNA and DNA (Supplementary table S4). Two types of AfAgo dimerization interfaces formed by the C-terminal β-sheets are observed in the structures. Both types of interfaces bury a comparable solvent-accessible surface area (Supplementary table S4), but result in a distinct arrangement of AfAgo subunits relative to one another, which we term “closed” and “open” dimers (Fig. 1a,b, respectively). The “closed” type of AfAgo homodimer, formed when the interface involves both the N-terminal residues and the C-terminal β-strands (Fig. 1a), provides a better fit to our SAXS data, suggesting that it is the major type of DNA-bound WT AfAgo dimer present in solution (Fig. 2c). It remains to be determined if the alternative “open” dimer observed in several structures (Fig. 1b and Supplementary table S4) was induced by crystal packing, or rather it is an alternative less abundant arrangement of AfAgo subunits that co-exists in solution at equilibrium with the “closed” form. As expected, removal of the β-strands located at the intersubunit interface (variant AfAgoΔ) impaired AfAgo dimerization (Fig. 2).

Simultaneous binding of WT AfAgo homodimer to both ends of a linear DNA fragment, one DNA end per AfAgo monomer, would result in a DNA loop. Formation of such looped DNA molecules upon incubation with WT AfAgo was directly visualized using AFM (Fig. 3). As shown in Table 1, ring-shaped AfAgo-DNA complexes constitute the majority of all protein–DNA complexes detected. Considerable decrease in the fraction of looped DNA complexes in the case of the dimerization interface mutant AfAgoΔ (Table 1) provides further proof that DNA looping is indeed mediated by the dimeric form of AfAgo protein.

To further characterize WT AfAgo–DNA interaction in solution, we have performed single-molecule FRET measurements (Fig. 4) using a DNA fragment labelled with fluorescent dye close to DNA ends. The design of the DNA substrate ensured that binding of WT AfAgo dimer to both DNA ends would bring the fluorophores into close proximity, resulting in FRET. Comparison of donor/acceptor channel records for free DNA and DNA with either WT AfAgo or dimerization-impaired AfAgoΔ confirmed that only WT AfAgo efficiently forms DNA loops, yet again implying that DNA looping is mediated by dimeric AfAgo.

**Figure 6:**
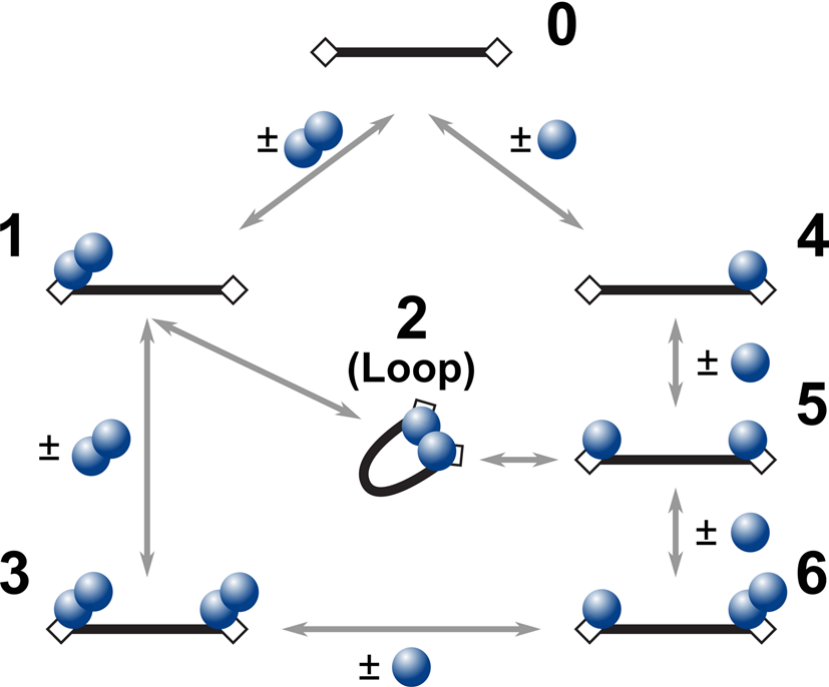
Kinetic schemes depicting possible reaction pathways between a two-target site DNA fragment and WT AfAgo. Black bars represent DNA, rectangular boxes—AfAgo-binding targets (phosphorylated DNA ends), blue circles—AfAgo monomers. Numbering of various protein–DNA assemblies matches numbering of complexes observed by AFM (Fig. 3). Note that species 1 and 6 may be formed via alternative pathways not depicted in the scheme (species 1 may be formed when two monomers associate consecutively with the same DNA end; species 6 may be formed when a monomer and a dimer associated with different DNA ends).

Assuming that wt AfAgo in its apo-form is an unstable dimer in solution (Fig. 2a), at least two types of mechanisms can be proposed for the formation of the WT AfAgo dimer / looped DNA complex, one involving apo-AfAgo homodimers (Fig. 6, left), the other involving apo-AfAgo monomers (Fig. 6**,** right). In the first scenario, the reaction may proceed via (i) association of free DNA (species “0”) with a single WT AfAgo dimer, which binds to one DNA end (species “1”); (ii) capture of the second DNA terminus by the DNA-bound AfAgo dimer in an intramolecular reaction, resulting in the looped complex (species “2”); (iii) alternatively, association of the second WT AfAgo dimer with the unoccupied second DNA end of species “1” leads to species “3”, which is no longer capable of loop formation. Such a mechanism was demonstrated for many proteins capable of DNA looping, including restriction endonucleases^35,39,40^ and transposases^41–44^. In the alternative scenario, DNA looping involves (i) binding of a single AfAgo monomer to the first DNA end (species “4”, Fig. 6); (ii) binding of the second AfAgo monomer to the second DNA end (species “5”); (iii) association of two DNA-bound monomers into the looped complex “2”; (iv) association of additional DNA-unbound AfAgo monomers with DNA-bound AfAgo subunits, a process that occludes loop formation (species “6” Fig. 6). Both these reaction pathways predict that at elevated protein concentrations the number of looped complexes should decrease due to simultaneous binding of separate AfAgo dimers to both DNA ends (species “3”). Single-molecule FRET experiments in solution support this prediction. Indeed, the amount of looped DNA molecules increases until an optimal protein concentration is reached (approx. 2 nM in our experimental setup, Fig. 4d), but declines upon further increase in WT AfAgo concentration. Despite the fact that all species depicted in Fig. 6 were detected using AFM (Fig. 3), the relative contribution of pathways involving WT AfAgo monomers and dimers into the overall DNA looping reaction remains to be established.

Single-molecule measurements on immobilized DNA allowed us to assess the dynamic properties of WT AfAgo-induced DNA loops. We find that (i) the DNA loops induced by WT AfAgo are relatively stable, with the lower limit estimate for the loop duration exceeding 30 s (Supplementary Fig. S8c); (ii) the proximity ratio E of the looped complexes changes over time, suggesting intrinsic dynamics of the AfAgo dimer attributable to the flexible dimerization interface (Supplementary Fig. S8d).

## Conclusions

The ability of WT AfAgo to form homodimers and bring two nucleic acid fragments into close proximity, to the best of our knowledge, was not previously described for any Argonaute protein. This finding underscores the diversity of prokaryotic Agos in general and broadens the range of currently known Argonaute-nucleic acid interaction mechanisms. In particular, it also raises questions regarding the currently unknown AfAgo function. Simultaneous interaction with two target sites in the case of restriction endonucleases is believed to increase specificity by preventing inadvertent cleavage of lone unmodified target sites^40,45^. Though AfAgo has no intrinsic nuclease activity, we cannot rule out its involvement (together with as of yet unidentified host nucleases) in host defence against invading nucleic acids, as recently proposed for the catalytically inactive full-length pAgo RsAgo^46^. The ability of WT AfAgo to form stable synaptic complexes with two DNA ends (in this study we used pre-formed blunt-end DNA substrates, though AfAgo likely first binds to the guide strand, and then recognizes an internal part of the target strand) is also reminiscent of transposases^41–44^, Cas1-Cas2 integrases^47,48^, and (retro)viral integrases^49,50^, which often bring the reactive 3′-OH groups of two DNA ends into proximity of the integration site. We therefore speculate that AfAgo could serve as a recognition module for the integrated DNA fragment; target DNA recognition, binding, and catalysis of the integration reactions would require involvement of additional, currently unknown, partner proteins. Intriguingly, some pAgos were recently shown to enhance homologous recombination in bacteria, a process that involves direct interaction of a catalytically active or inactive pAgo PIWI domain with RecA recombinase^51^. It remains to be seen if homologous recombination is also enhanced by AfAgo, and if it is affected by AfAgo dimerization. We currently continue structural–functional studies of AfAgo and related short prokaryotic Argonautes, primarily focusing on the identification of their partner proteins and possible biological roles.

## Supplementary Information

Supplementary Information 1.

Supplementary Information 2.

## Data Availability

*Accession codes A. fulgidus* (ATCC 49558) AfAgo, UniProtKB entry O28951.
