# Supplementary material for: Prokaryotic Argonaute from Archaeoglobus fulgidus interacts with DNA as a homodimer
Source: Sci Rep. 2021 Feb 25;11:4518. doi: 10.1038/s41598-021-83889-4 (PMC7907199; doi:10.1038/s41598-021-83889-4)
Supplement: Supplementary file 1 — Supplementary Information 1. [file 41598_2021_83889_MOESM1_ESM.pdf]

SUPPLEMENTARY DATA

Prokaryotic Argonaute from *Archaeoglobus fulgidus* interacts with DNA as a homodimer

Edvardas Golovinas[1], Danielis Rutkauskas[1,2], Elena Manakova[1], Marija Jankunec[1,3], Arunas Silanskas[1], Giedrius Sasnauskas[1], Mindaugas Zaremba[1,]*

[1] Institute of Biotechnology, Life Sciences Center, Vilnius University, Sauletekio av. 7, LT-10257, Vilnius, Lithuania.

[2] Institute of Physics, Center for Physical Sciences and Technology, Savanoriu 231, LT-02300, Vilnius, Lithuania

[3] Institute of Biochemistry, Life Sciences Center, Vilnius University, Sauletekio av. 7, LT-10257, Vilnius, Lithuania.

* To whom correspondence should be addressed. Tel: +370-5-2234357; Fax: +370-5-2234367; Email: zare@ibt.lt.

Volumetric analysis

The stoichiometry of both analysed proteins was assessed from volumetric analysis. The molecular volume of protein was determined by measuring the height and half-height diameters of two perpendicular cross-sections. The particle is treated as a spherical cap and the volume of each protein particle was calculated according to Eq. 1:

$$V_m = (\frac{\pi h}{6})(3r^2 + h^2) \qquad \text{(Eq. 1)},$$

where h is the particle height and r is the radius at half height [1]. Molecular volume based on molecular weight was calculated using the equation:

$$V_c = (\frac{M_0}{N_0})(V_1 + dV_2) \qquad \text{(Eq. 2)},$$

where M_0 is the molecular mass of the protein, N_0 is Avogadro's number, V_1 and V_2 are the partial specific volumes of protein and water (0.74 cm^3g^{-1} and 1 cm^3g^{-1}, respectively), and d is the extent of protein hydration (0.4 mol water/mol protein) [2]. Thus, the calculated volume of WT AfAgo (50.8 kDa) and AfAgoΔ (49.9 kDa) proteins is *approx.* 100 nm^3. The measured volume data was divided into three populations by their theoretical volume: *monomer* (<150 nm^3), *dimer* (150 – 250 nm^3), *higher-order* (>250 nm^3). Data are summarized in Table 1. Supplementary figure S5 shows no correlation between the measured volume of bound protein and the measured DNA contour length.

Single-molecule setup

We used a custom single-molecule fluorescence microscopy setup built on a commercial inverted microscope Nikon Eclipse Ti-U equipped with 60× 1.2 WI Plan Apo VC objective (Nikon) used for the excitation and signal collection, two avalanche photodiode (APD)-based single-photon counting modules (Tau-SPAD-50, PicoQuant) and 25 mW 532 and 635 nm diode-pumped solid-state and diode lasers (Crystalaser), respectively. The laser excitation was reflected

off a dichroic mirror (zt532/635rpc-XT, Chroma), and the fluorescence signal filtered off the excitation light with a quadruple-band interference filter (FF01-446/510/581/703, Semrock) and split into two spectral channels with a dichroic mirror (645dcxr, Chroma). ALEX was implemented by directly TTL-modulating the intensity of the 635 nm laser and synchronously modulating the intensity of the 532 nm laser with a mechanical chopper (MC2000B, Thorlabs). The half period of ALEX was 50 μs. Fluorescence photon arrival times were recorded and ALEX was implemented using an FPGA module (PCIe-7851R, National Instruments) and custom Labview (National Instruments) program.

The excitation was focused 50 μm above the sample chamber glass surface. 532 nm excitation intensity was 30 μW, 635 nm - 20 μW. The size of the confocal pinhole was 75 μm. Each measurement was 10 min long.

Single-Molecule Data analysis

Fluorescence burst analysis was performed using the freely available FRETBursts software [3]. The initial bursts search parameters were m = 10 photons, and F = 6 times the fluorescence background. The total intensity of a burst from both channels and excitation wavelengths was thresholded to be larger than 40 counts, and this yielded ~3000 bursts from a 10 min measurement. Each burst was calculated a proximity ratio, E, according to $E = I_d^a/(I_d^a + I_d^d)$, here I_d^a and I_d^d are acceptor and donor intensities upon donor excitation, respectively, and stoichiometry parameter, S, according to $S = I_d/(I_d + I_a^a)$, here I_d is the total donor and acceptor intensity upon donor excitation, and I_a^a is acceptor intensity upon acceptor excitation. Then we built 2D E-S histograms of bursts. Subsequently, bursts with stoichiometry parameter ranging from 0.2-0.9 were selected to build distributions of the proximity ratio, E, of bursts of DNA molecules labelled with both fluorophores only. E histograms were fit with the sum of two

Gaussian functions using unconstrained optimization. Then the ratio of the number of looped and unlooped DNA molecules in the ensemble was calculated as the ratio of the area of the Gaussian of high E with that of low E.

To quantify the looped state duration the E trajectories were idealized using HMM with a two-state model in QuB software [4]. Then, the cumulative histogram of the looped state durations was built from the idealized trajectories. The trajectory edge dwells were not omitted in order to preserve the information on the occurrence of states lasting during the whole trajectory. The exponential factor of a single-exponential fit of the cumulative histogram was 33±1 s. The maximum recorded looped state duration is, however, limited by the duration of our measurement (200 s) and the duration of the fluorescent state of the fluorophores before photobleaching. The value of the exponential factor thus sets the lower limit of the looped state duration.

The experiment of surface-immobilized DNA fragments was done by first recording a short movie with 635 nm excitation to obtain a reference for fluorescent spot identification since the acceptor channel exhibits significantly less fluorescence background than the donor channel. Then a longer actual movie was recorded with the 532 nm excitation. The analysis of the two-spectral channel fluorescence movies was performed using custom software written in Matlab. Briefly, to identify the fluorescent spots, the first 20 frames of the reference and the actual fluorescence movies were averaged, the obtained average images were filtered with the 2D low-pass Gaussian filter 5 pixels large and with the standard deviation of 1 pixel and subtracted the same image filtered with the averaging filter 7 pixels large. The resulting acceptor channel reference image was thresholded with 20 and the donor channel actual image - with 40 counts/pixel. The obtained images were binarized for particle identification. Particles in both

binary images were identified and filtered according to the following criteria: 5x5 pixel ROIs (regions of interest) centred on particles' centres of mass had to non-overlap, particle area had to be within 5-100 pixels range, particle eccentricity not larger than 0.8. The coordinates of a particle in the donor channel corresponding to a particle identified in the acceptor channel of the reference movie were calculated using the spatial transformation structure calculated from an image of surface-immobilized 200 nm fluorescent polystyrene beads (F8806, Invitrogen). For trace extraction were considered only those particles in the actual movie whose donor coordinates coincided with the transformed coordinates of the acceptor particles in the reference movie within 1.5 pixels. The donor and acceptor intensity traces were extracted using aperture photometry [5] with the background calculated as an average intensity from a 1 pixel-wide annulus around the particle's ROI. The proximity ratio, E, was calculated according to the same formula as for the fluorescence bursts.

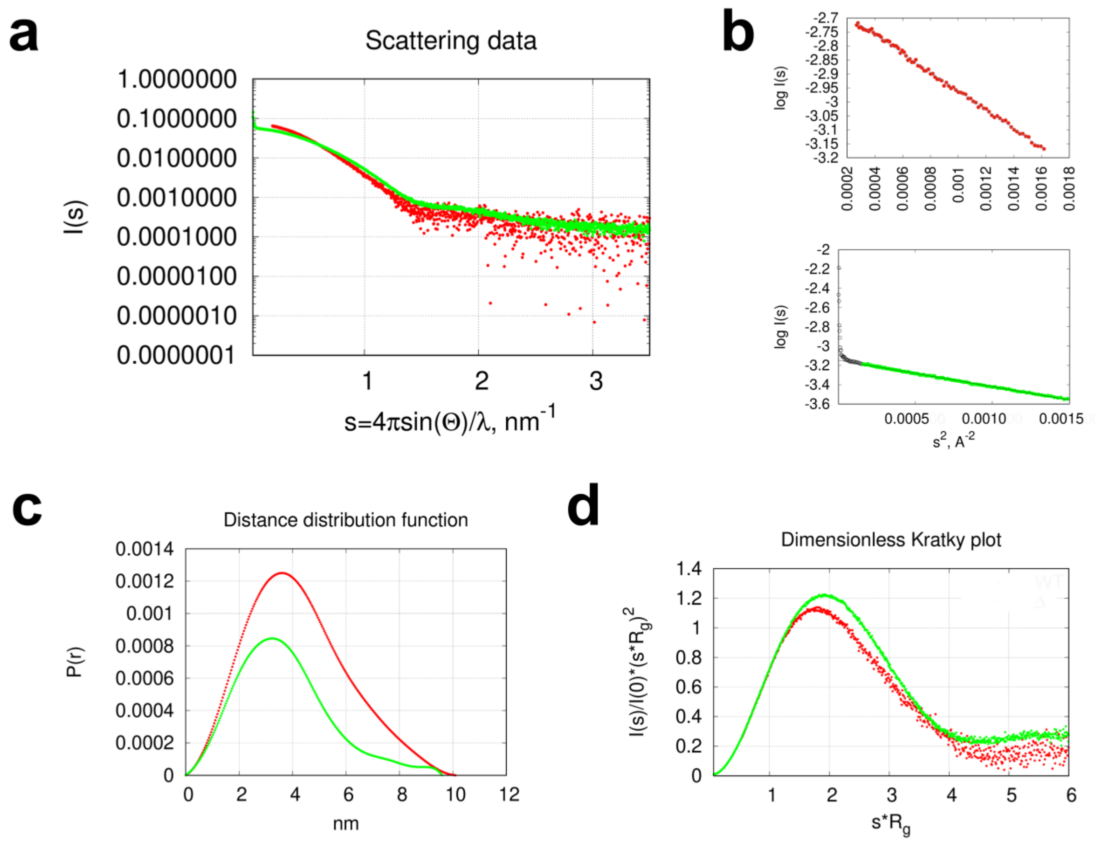

Supplementary figure S1. SAXS data of AfAgo+MZ-1289 (red curves) and monomeric mutant AfAgoΔ+MZ-1289 (green curves) complexes. (**a**), Scattering curves. (**b**), Guinier plots log I(s) vs. s^2 of the data at small s values. (**c**), Pair distance distribution functions. (**d**), Dimensionless Kratky representation of scattering data $(I(s)/I(0)*(s*Rg)^2$ vs. s*Rg). All curves have similar shapes typical for folded proteins [6].

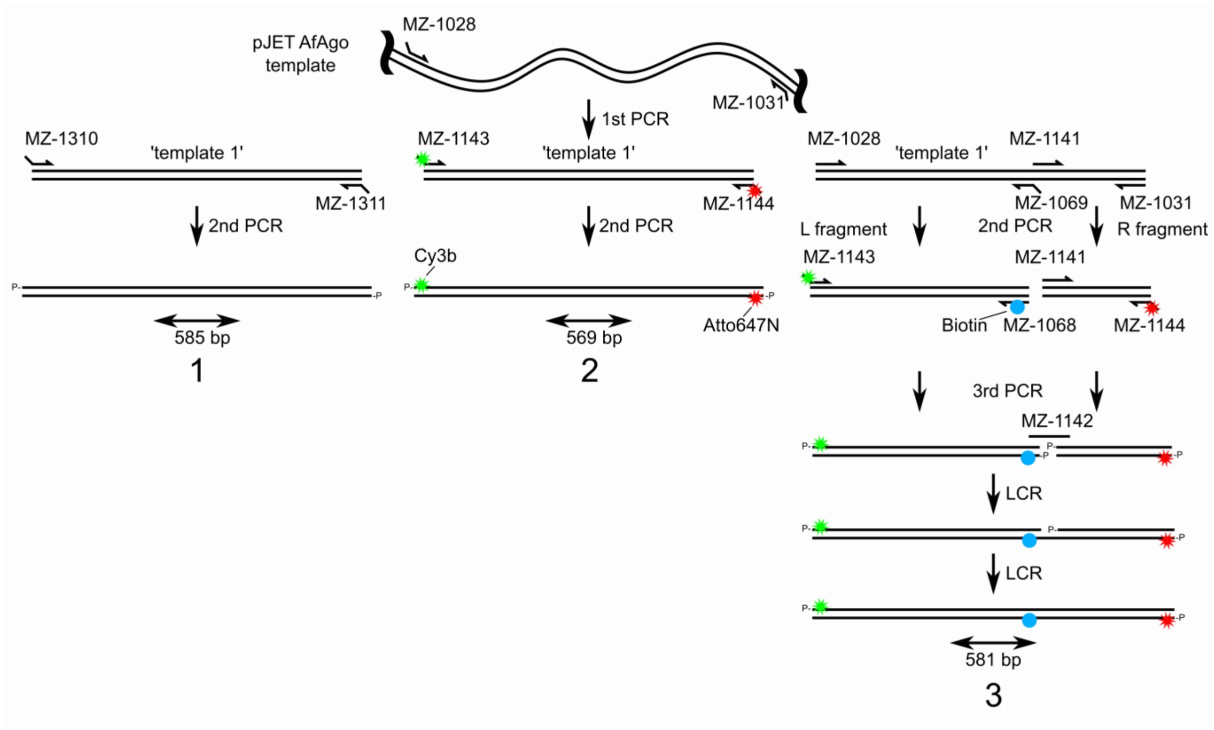

Supplementary figure S2. Synthesis scheme for the DNA fragments. First, a DNA fragment was amplified from a pJET plasmid template containing an AfAgo gene fragment using oligonucleotides MZ-1028 and MZ-1031. The PCR product was then used as a template (dubbed "template 1") in subsequent reactions. Fragment "1" used for AFM studies was made by PCR from "template 1", using oligonucleotides MZ-1310 and MZ-1311, which were treated with T4 polynucleotide kinase (PNK) prior to amplification, to yield a 585 bp fragment. Fragment "2" was amplified from "template 1" with oligonucleotides MZ-1143 and MZ-1144, bearing Cy3B (green star) and Atto647N (red star) dyes, respectively, on the third base from the 5'-end, yielding 569 bp DNA. Fragment "3" was synthesised in two steps. Firstly, respective fragments flanking the biotinylation site (dubbed "L fragment" and "R fragment") were amplified by PCR from "template 1", using primer pairs MZ-1028 and MZ-1069 for the "L fragment", and MZ-1031 and MZ-1141 for the "R fragment". Secondly, each of the two fragments was used as templates for subsequent PCRs. "L fragment" was amplified using MZ-1143 and MZ-1068, the latter bearing the biotin (blue circle) on 22 b from its 5'-end. "R fragment" was amplified using primers MZ-1141 and MZ-1144. The two fragments were then purified using a GeneJET PCR purification kit (ThermoFisher Scientific) and treated with PNK while mixed in equal amounts to a total concentration of 6 nM. The phosphorylation mix was subsequently ligated by Ampligase®

(Epicentre, USA) at 3 nM total DNA and 30 nM bridging oligonucleotide MZ-1142 according to Chandran, 2017 [7]. All full-length DNA fragments were subsequently purified from an agarose gel using a runVIEW system (Cleaver Scientific, UK), precipitated with sodium acetate/isopropanol, washed with 75% ethanol, and resuspended in water.

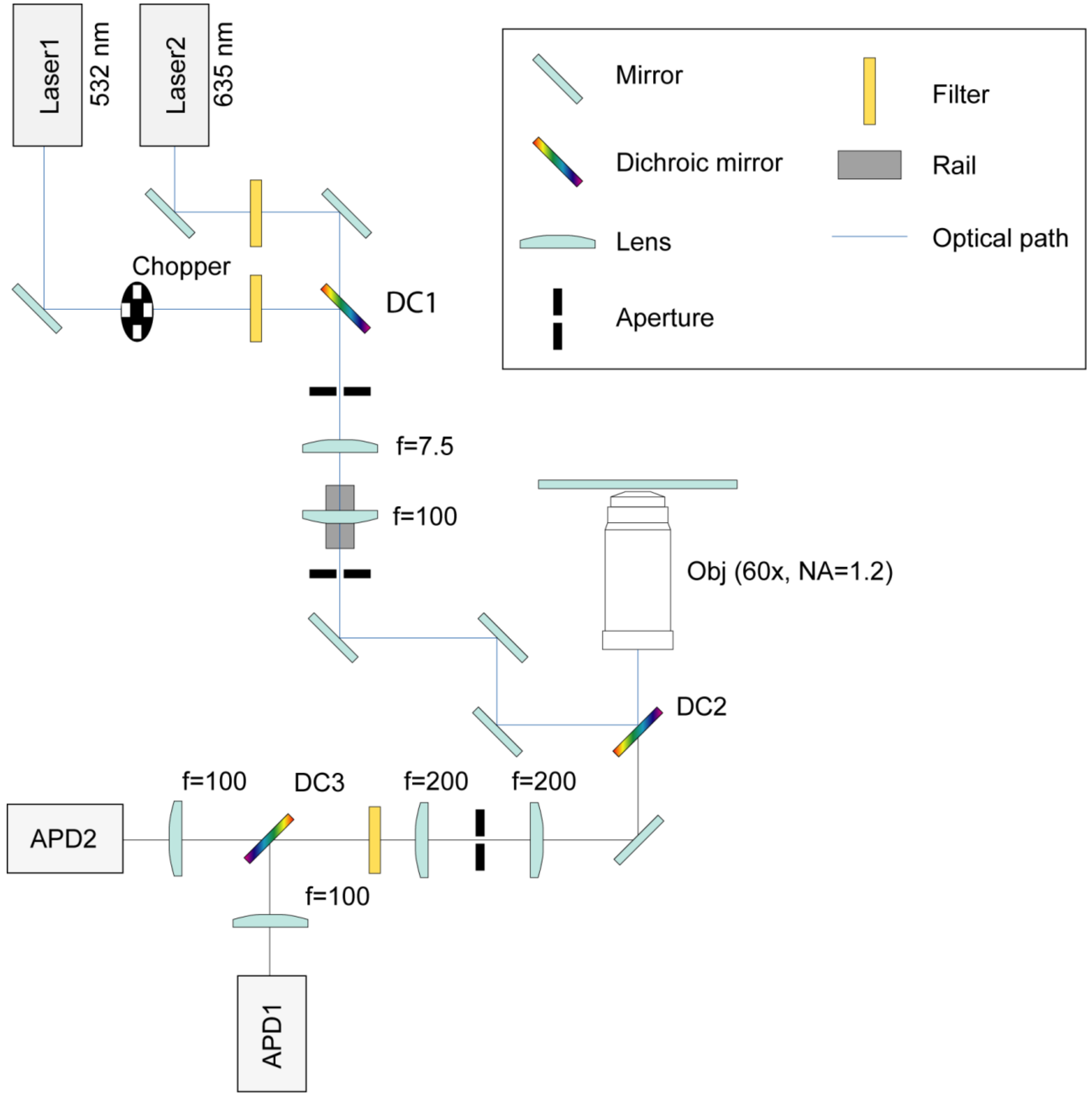

Supplementary figure S3. Optical scheme of custom single-molecule fluorescence microscopy setup used to record fluorescence bursts of single diffusing molecules in this study. APD – avalanche photodiode; f – focal distance; NA – numerical aperture.

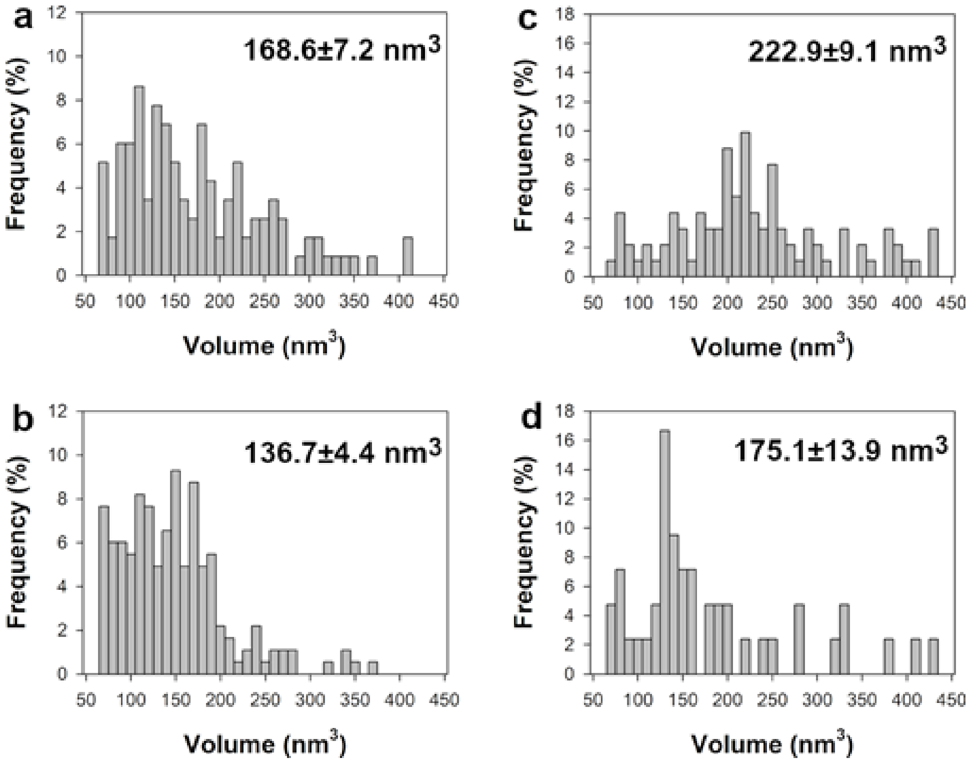

Supplementary figure S4. Volumetric analysis of WT AfAgo (panels **a** and **c**) and AfAgoΔ (panels **b** and **d**) proteins in protein-DNA complexes observed by AFM. Volumes of proteins bound to ends of unlooped DNA molecules for WT AfAgo (n=118) and AfAgoΔ (n=183) complexes are presented in panels **a** and **b**, respectively. Volumes of proteins bound to looped DNA for WT AfAgo (n=95) and (**d**) AfAgoΔ (n=44) complexes are shown in panels **c** and **d**. Mean ± S.E.M. values for each set are shown.

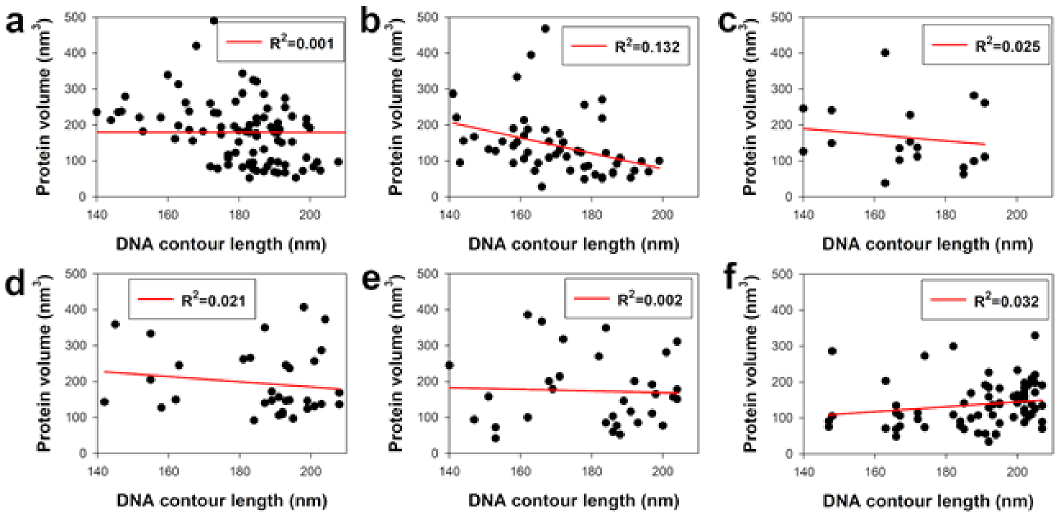

Supplementary figure S5. Correlation between the bound protein volume and the DNA contour length in AfAgo-DNA complexes as measured by AFM. WT AfAgo-DNA complexes: (**a**) protein bound to looped DNA (n=90), (b) protein bound to one end of linear DNA (n=53), and (c) protein bound to both ends of linear DNA (n=18); AfAgoΔ complexes: (d) protein bound to looped DNA (n=34), (e) protein bound to one end of linear DNA (n=32), and (f) protein bound to both ends of linear DNA (n=72).

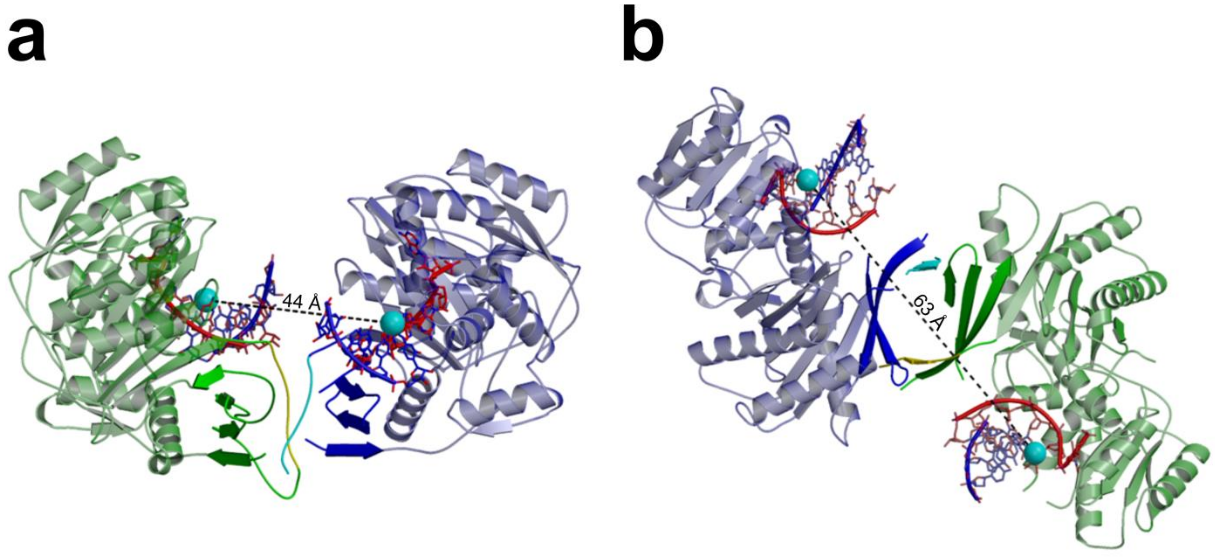

Supplementary figure S6. Expected positions of fluorescent labels upon formation of the looped complex. The figure is based on PDB ID 1ytu (**a**, “closed”), 2w42 (**b**, “open”), spheres mark fluorophore attachment sites. Protein monomers are coloured green and blue, DNA guide and target strands are red and blue respectively.

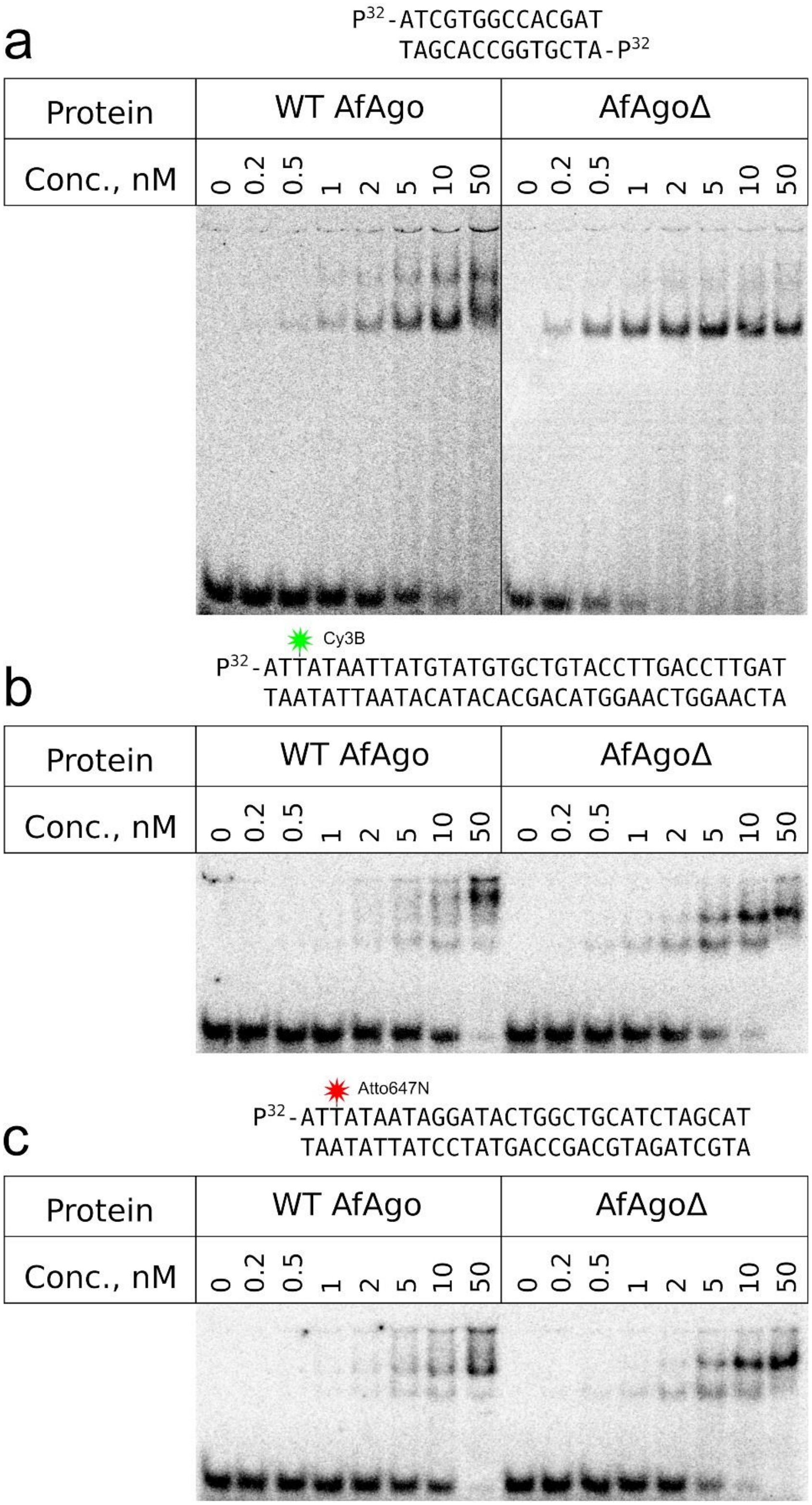

Supplementary figure S7. DNA binding by AfAgo. DNA binding was verified using electrophoretic mobility shift assay. 5′P^{32}-labelled DNA substrates were: a self-complementary

oligoduplex MZ-952 (panel a); a Cy3B-modified oligoduplex MZ-1443/MZ-1026 (panel b); a Atto647N-modified oligoduplex MZ-1144/MZ-1027 oligoduplex (panel c). The fluorescently-modified oligoduplexes carried the 5′-P^{32} label only on the modified strand. DNA concentration in the binding reactions was 1 nM, final protein concentrations are shown above each lane. The binding buffer was 40 mM Tris-acetate (pH 8.4 at 25 °C) with 1 mM EDTA (TAE, B49, Thermo Scientific), supplemented with 5 mM $(CH_3COO)_2Mg$, 0.1 mg/ml BSA, 1 mM DTT, 10% (v/v) glycerol. Running buffer – TAE (Thermo Scientific) supplemented with 5 mM $(CH_3COO)_2Mg$.

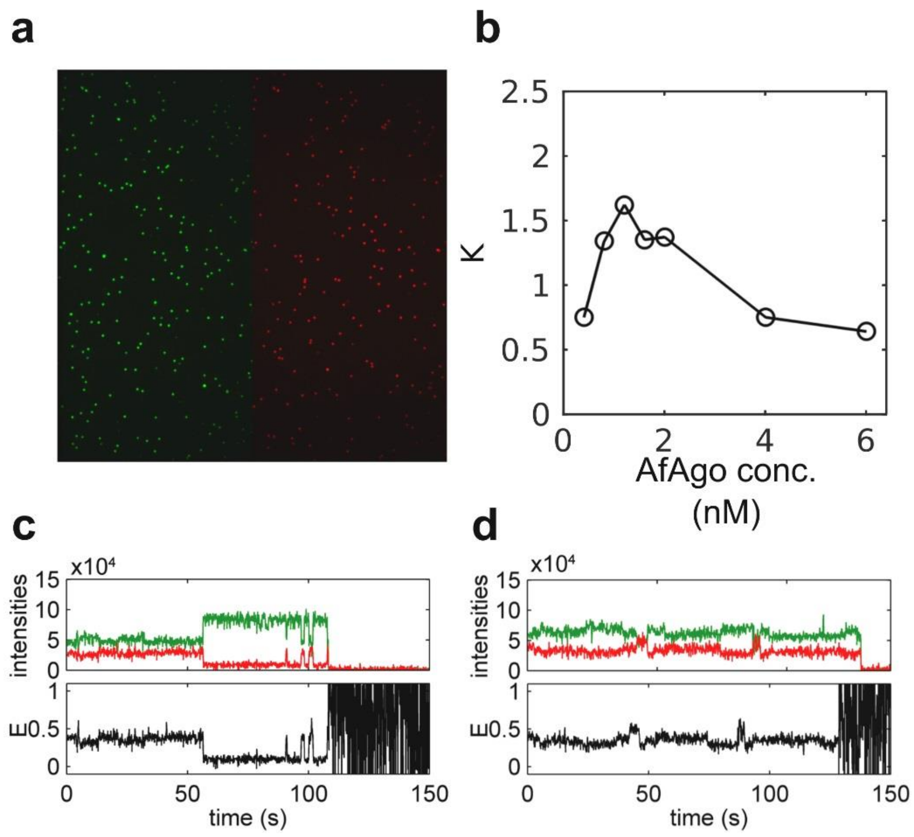

Supplementary figure S8. Single-molecule experiments. (**a**) A fluorescence image of surface-immobilized DNA fragments. It is an average of 20 frames in a fluorescence movie. The left part (green) is the donor image upon donor excitation, and the right part (red) is the acceptor image upon acceptor excitation. (**b**) The dependence of the ratio, K, of the number of looped and unlooped DNA molecules depending on the concentration of the AfAgo for the biotinylated DNA fragment in solution. (**c**, **d**) Examples of different dynamics of DNA looping by AfAgo in TIRF experiments.

Supplementary table S1. List of oligonucleotides used in this study.

Oligonucleotide	Sequence, 5‘->3‘	Modifications
MZ-383	TGATTCTGCAGTTATAGGAACCACGGATTCGTTTGTAATGAGC	
MZ-385	TGATTGGATCCGATGATGGAATATAAAATAGTTGAAAATGGTTTGAC	
MZ-875	GCTATACTTCACTTAAATGAAACTCCTAACAATAGATTTCATCCGTATG	
MZ-876	CCTTCATACGGATGAAATCTATTGTTAGGAGTTTCATTTAAGTGAAGTATAGC	
MZ-952	ATCGTGGCCACGAT	
MZ-1026	ATCAAGGTCAAGGTACAGCACATACATAATTATAAT	
MZ-1027	ATGCTAGATGCAGCCAGTATCCTATTATAAT	
MZ-1028	GTGCTGTACCTTGACCTTGATGAACTGGCGCAACACGTATTG	
MZ-1031	ATACTGGCTGCATCTAGCATACGATCTCAACACTTAATGGTTT	
MZ-1068	ATTCTGGTCTCGGACTCCCATTACCCAAAATGGATGAG	Biotin on T22
MZ-1069	ATTCTGGTCTCGGACTCCCATTACCCAAAATGGATGAG	
MZ-1141	CCTAACAATAGATTTCATCCG	
MZ-1142	GGGTAATGGGAGTCCGAGACCAGAATCCTAACAATAGATTTCATCCGTATGAAGG	
MZ-1143	ATTATAATTATGTATGTGCTGTACCTTGACCTTGAT	Cy3b on T3, 5‘P
MZ-1144	ATTATAATAGGATACTGGCTGCATCTAGCAT	Atto647N on T3, 5‘P
MZ-1310	ATTGCTCTACTGTATAATGCTGTGCTGTACCTTGACCTTGAT	

MZ-1311	ATTGCTCTACTGTATAATGCTATACTGGCTGCATCTAGCAT	
MZ-1289	ATTGTACGTACAAT	5‘P

Supplementary table S2. SAXS data collection and main structural parameters

Instrument, Detector	P12, pilatus6m	
Detector-to-sample distance, m	3.0	
Wavelength, nm	0.123981	
Measured s range, nm^{-1}	0.0224526-7.3176000	
Number of buffer exposure frames averaged (measured) / frame exposure time	101 (101) / 0.995 sec	76 (80) / 0.195 sec
Number of sample exposure frames averaged (measured) / frame exposure time	24 (24) / 0.995 sec	30 (40) / 0.195 sec
Capillary temperature/ Sample changer temperature	20 °C / Room temperature	20 °C / 10 °C
Data reduction and on-line characterization	radaver (r11095), databsolute v0.1 (r11095)	
Structural parameters		
Sample	WT AfAgo+MZ-1289, SEC peak	AfAgoΔ+MZ-1289, 4 mg/ml
Guinier points (AUTORG)	1-87	39-132
s range, nm^{-1} (points) used in GNOM	0.0640-3.3457 (1-1200)	0.1860-3.3457 (60-1200)
Rg, nm (AUTORG/ GNOM)	3.18 ± 0.016/ 3.233 ± 0.005202	2.84 ± 0.03/ 2.879 ± 0.002440
I(0) (AUTORG/ GNOM)	0.0725 ± 0.00011/ 0.07301 ± 0.00008771	0.0428 ± 3.7e-05/ 0.04289 ± 0.00002499
Dmax, nm (DATCLASS/ SHANUM/ GNOM)	11.3/ 10.2/ 10.1	10.9/ 10.5/ 9.6

Porod volume, nm^3 (DATPOROD)	158.03	108.67
SASBDB ID	SASDH39	SASDH49

Supplementary table S3. Molecular mass determination from SAXS data using various methods. All molecular masses are given in kDa

Sample			WT AfAgo+MZ-1289	AfAgoΔ+MZ-1289
Expected M_w (protein + DNA), kDa			119	58.6
Method	Reference	Sofware	MWcalc	
Absolute scale	[8]	PRIMUS 2.8.4 (r10552)	99.7	55.4
Qp			102.7	58.5
Bayes			94.2	56.9
Size&Shape			100.0	67.9
Porod volume/1.6	[9]	DATPOROD, ATSAS 2.8.4 (r10552)	98.8	67.9
SAXSMoW	[10]	SAXSMoW v2.1 http://saxs.ifsc.usp.br/	106.9 (integrated to I0/I(qmax)=102.25)	67.4 (integrated to I0/I(qmax)=102.25)
SEC MW		CHROMIXS ATSAS 2.8.4 (r10552)	103.8	n.a.

Supplementary table S4. AfAgo dimerization interfaces as analyzed by PISA (PDBe PISA v1.52 [20/10/2014])

PDB ID	Dimer: open/ closed	Image	CSS Complex Formation Significance Score	ΔiG P-values	PISA: dimerization surface, $Å^2$ (buried in interface)
1w9h	open	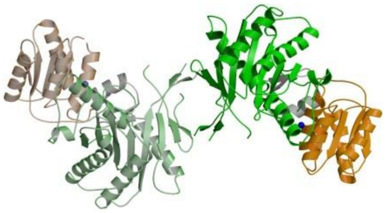	0.108 *	0.004	731
1ytu	closed	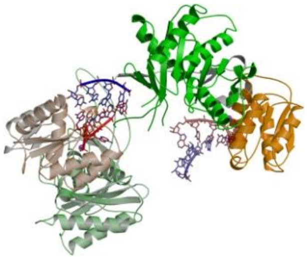	1	0	908
2bgg	open	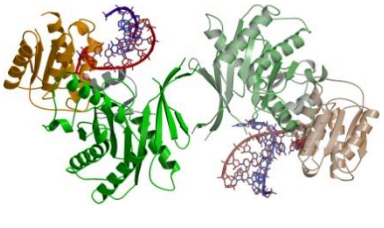	1	0.001	601
2w42	open	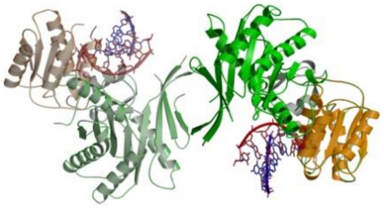	1	0.002	748

* The dimerization interface in PDB ID 1w9h is essentially identical to interfaces in PDB IDs 2bgg and 2w42. The lower CSS score is due to the fact that PISA gives lower scores to interfaces generated by symmetry operators (as is in the case of PDB ID 1w9h, which contains a single AfAgo subunit per asymmetric unit) than to interfaces formed between different subunits present in the asymmetric unit (the dimers in PDB IDs 2bgg and 2w42 are formed by 2 AfAgo monomers present in the asymmetric unit).

Supplementary Information References

1. Schneider, S. W., Lärmer, J., Henderson, R. M. & Oberleithner, H. Molecular weights of individual proteins correlate with molecular volumes measured by atomic force microscopy. *Pflugers Arch. Eur. J. Physiol.* **435**, 362–367 (1998).

2. Edstrom, R. D. *et al.* Direct visualization of phosphorylase-phosphorylase kinase complexes by scanning tunneling and atomic force microscopy. *Biophys. J.* **58**, 1437–1448 (1990).

3. Ingargiola, A., Lerner, E., Chung, S. Y., Weiss, S. & Michalet, X. FRETBursts: An open source toolkit for analysis of freely-diffusing Single-molecule FRET. *PLoS One* **11**, 1–27 (2016).

4. Nicolai, C. & Sachs, F. Solving ion channel kinetics with the QuB software. *Biophys. Rev. Lett.* **8**, 191–211 (2013).

5. Holden, S. J. *et al.* Defining the Limits of Single-Molecule FRET Resolution in TIRF Microscopy. *Biophys. J.* **99**, 3102–3111 (2010).

6. Durand, D. *et al.* NADPH oxidase activator p67phox behaves in solution as a multidomain protein with semi-flexible linkers. *J. Struct. Biol.* **169**, 45–53 (2010).

7. Chandran, S. Rapid Assembly of DNA via Ligase Cycling Reaction (LCR). in *Synthetic DNA: Methods and Protocols* (ed. Hughes, R. A.) 105–110 (Springer New York, 2017). doi:10.1007/978-1-4939-6343-0_8.

8. Konarev, P. V., Volkov, V. V., Sokolova, A. V., Koch, M. H. J. & Svergun, D. I. PRIMUS - a Windows-PC based system for small-angle scattering data analysis. *J. Appl. Crystallogr.* **36**, 1277–1282 (2003).

9. Petoukhov, M. V. *et al.* New developments in the ATSAS program package for small-angle scattering data analysis. *J. Appl. Crystallogr.* **45**, 342–350 (2012).

10. Fischer, H., De Oliveira Neto, M., Napolitano, H. B., Polikarpov, I. & Craievich, A. F. Determination of the molecular weight of proteins in solution from a single small-angle

X-ray scattering measurement on a relative scale. *J. Appl. Crystallogr.* **43**, 101–109 (2010).
