# Supplementary material for: Prokaryotic Argonaute from Archaeoglobus fulgidus interacts with DNA as a homodimer
Source: Sci Rep. 2021 Feb 25;11:4518. doi: 10.1038/s41598-021-83889-4 (PMC7907199; doi:10.1038/s41598-021-83889-4)
Supplement: Supplementary file 2 — Supplementary Information 2. [file 41598_2021_83889_MOESM2_ESM.docx]

SUPPLEMENTARY DATA

Single-molecule FRET setup and data analysis, SAXS data, DNA fragment synthesis scheme, the optical scheme of single-molecule setup, expected fluorescent label positions in AfAgo-DNA complex, EMSA results, additional single-molecule FRET results, list of oligonucleotides, SAXS data collection and structural parameters, SAXS molecular mass determination, and dimerization surface analysis by PISA (PDF).
